# Transcriptional and Post-Transcriptional Regulation of Thrombospondin-1 Expression: A Computational Model

**DOI:** 10.1371/journal.pcbi.1005272

**Published:** 2017-01-03

**Authors:** Chen Zhao, Jeffrey S. Isenberg, Aleksander S. Popel

**Affiliations:** 1 Department of Biomedical Engineering, School of Medicine, Johns Hopkins University, Baltimore, Maryland, United States of America; 2 Vascular Medicine Institute, Division of Pulmonary, Allergy, and Critical Care Medicine, Department of Medicine, University of Pittsburgh, Pittsburgh, Pennsylvania, United States of America; University of Virginia, UNITED STATES

## Abstract

Hypoxia is an important physiological stress signal that drives angiogenesis, the formation of new blood vessels. Besides an increase in the production of pro-angiogenic signals such as vascular endothelial growth factor (VEGF), hypoxia also stimulates the production of anti-angiogenic signals. Thrombospondin-1 (TSP-1) is one of the anti-angiogenic factors whose synthesis is driven by hypoxia. Cellular synthesis of TSP-1 is tightly regulated by different intermediate biomolecules including proteins that interact with hypoxia-inducible factors (HIFs), transcription factors that are activated by receptor and intracellular signaling, and microRNAs which are small non-coding RNA molecules that function in post-transcriptional modification of gene expression. Here we present a computational model that describes the mechanistic interactions between intracellular biomolecules and cooperation between signaling pathways that together make up the complex network of TSP-1 regulation both at the transcriptional and post-transcriptional level. Assisted by the model, we conduct *in silico* experiments to compare the efficacy of different therapeutic strategies designed to modulate TSP-1 synthesis in conditions that simulate tumor and peripheral arterial disease microenvironment. We conclude that TSP-1 production in endothelial cells depends on not only the availability of certain growth factors but also the fine-tuned signaling cascades that are initiated by hypoxia.

## Author Summary

Research evidence show that thrombospondin-1 (TSP-1) is an anti-angiogenic protein which potently inhibits the downstream signaling of vascular endothelial growth factor receptor 2 (VEGFR2), an important pathway that promotes endothelial cell proliferation, migration and permeability. As demonstrated by numerous studies, expression of TSP-1 is often upregulated in peripheral arterial disease (PAD) and downregulated in many solid tumors, primarily because of its inhibitory effect on angiogenesis and tumor growth. Given the established anti-angiogenic property of TSP-1 and its dysregulation in diseases, it holds great value to design novel therapeutic strategies that aim to restore TSP-1 expression in tumors and limit its expression in PAD by regulating the biomolecules that control TSP-1 synthesis. The computational, mechanistic, experiment-based model of TSP-1 intracellular regulation presented here is a solid integration of the current knowledge and is substantially validated against published data. Our model simulations reproduce the experimental time-course dynamics of key proteins within the regulatory network and suggest interesting behavior in hypoxia- and cytokine-driven regulation of TSP-1. In addition, we assess different model-based strategies to modulate TSP-1 synthesis *in silico*; our model is the essential module of an integrated computational platform that can provide research insights for future investigations of TSP-1 and angiogenesis.

## Introduction

The growth of tumor depends on its surrounding vascular supply, which is commonly stimulated by the overexpression of tumor-secreted pro-angiogenic factors including VEGF [1]. Given the importance of the pro-angiogenic pathway downstream of VEGF, inhibiting the VEGF signaling axis has proven an effective therapy for patients with solid tumors and neovascular age-related macular degeneration; drugs such as bevacizumab and aflibercept which sequester circulating VEGF have shown efficacy in some of these clinical situations [1, 2]. Research has also found that human and animals can produce various anti-angiogenic molecules. An example of a potent endogenous anti-angiogenic protein is thrombospondin-1 (TSP-1) [3]. TSP-1 was the first protein identified as a naturally occurring inhibitor of angiogenesis. It is a large matricellular protein that interacts with various ligands and receptors, including components of the extracellular matrix, growth factors, cell surface receptors and cytokines [4]. One of the major pathways that TSP-1 negatively regulates to inhibit angiogenesis is the VEGF-VEGFR2 axis. It is reported that secreted TSP-1 binds to its high affinity receptor CD47 and disrupts the association of VEGFR2 with CD47, thereby downregulating the pro-angiogenic signals downstream of VEGF; another mechanism proposed to explain the inhibitory effect of TSP-1 on VEGF-mediated angiogenesis involves the TSP-1 receptor CD36 and endothelial cell apoptosis pathways [5]. Although the detailed mechanism of action of TSP-1 as an anti-angiogenic protein is not fully understood, the potential of TSP-1 and its analogs as therapeutics against cancer has already been demonstrated by several preclinical and clinical studies [6–9].

The expression of TSP-1 in tumors is often found dysregulated. In some tumors with negative TSP-1 expression, tumor vascularity is significantly higher and this is associated with worse prognosis than patients with TSP-1 positive tumors [10, 11]. Because of its strong anti-angiogenic effect, the role of TSP-1 in ischemic vascular diseases has also been investigated. Interestingly, in the plasma and tissue samples collected from patients with peripheral arterial disease (PAD), TSP-1 is highly upregulated [12, 13]. Hypoxia is also reported to increase TSP-1 synthesis in non-tumor conditions in various cell types including endothelial cells (ECs), fibroblasts, renal tubular epithelial cells and vascular smooth muscle cells [14–17]. This effect may be parallel to the induction of VEGF in hypoxic conditions, suggesting a potential negative feedback loop that limits angiogenesis in certain conditions.

Besides the direct intervention of TSP-1/VEGFR/CD47 interactions on the cell surface, another potential therapeutic strategy to harvest the anti-angiogenic potential of TSP-1 that is underexplored is the modulation of its intracellular synthesis [5]. In addition to the transcriptional regulation by promoters and repressors such as HIF-2α and Myc, TSP-1 expression is also tightly regulated by several microRNAs including miR-18a [14, 18–20]. The HIF-let7-AGO1 pathway is shown to limit microRNA biogenesis in hypoxic conditions and is likely a contributing factor to the downregulation of miR-18a in hypoxia [21–23]. The abundance of miR-18a is also regulated by Myc while Myc expression is repressed by HIF-1 through multiple mechanisms [24, 25]. Therefore, formulating and analyzing the signaling axis that connects HIF, Myc, microRNA and TSP-1 in hypoxia may provide insights into the complex dynamics of TSP-1 induction and help screen therapeutic strategies that can efficiently modulate TSP-1 synthesis to regulate angiogenesis.

TSP-1 can activate the latent TGFβ (transforming growth factor beta) molecule, a multifunctional cytokine that plays a key role in inflammation, wound healing, cell proliferation and immune response [26]. The ligand TGFβ promotes the synthesis of TSP-1 via a positive feedback, possibly through downstream SMAD signals [27]. Another possible mechanism of how TGFβ mediates TSP-1 synthesis is through the influx of calcium upon TGFβ ligation and the subsequent calcium-mediated activation of NFATc1 (nuclear factor of activated T-cells 1) which is found to be a TSP-1 promoter [28, 29]. Summarizing both the hypoxic and TGFβ stimulation of intracellular TSP-1, our mechanistic model presented in this study is the first computational model that considers pathway interactions between the different modes of TSP-1 regulation discussed above. Previous models of TSP-1 studied its interaction with receptors on the cell membrane or TGF-β in the extracellular matrix and paid minimal attention to the complex story of TSP-1 regulation within the cell, but we consider it very relevant to TSP-1 dysregulation in diseases [30, 31]. Thus the focus of this work is restricted to hypoxia- and TGFβ-mediated pathways that regulate TSP-1 expression in ECs. We also explored the potential application of the model in more than one cell type, because of the fact that different groups of cells might be responsible for the synthesis of TSP-1 in different pathological conditions [32]. Assisted by the model, we have identified several key characteristics of intracellular TSP-1 regulation, focusing on the interactive signaling events during receptor activation and hypoxia, as well as the hierarchical regulation of TSP-1 mRNA orchestrated by different intermediate species and microRNAs. We also simulated the model under selected conditions that mimic certain protein profiles observed in tumors and PAD and tested different therapeutic interventions to restore the dysregulated TSP-1 expression back to baseline. The findings presented in this study should help design future experimental and computational research to further investigate the mechanistic regulatory networks that contribute to the abnormal TSP-1 expressions in cancer and in ischemic vascular disease.

## Results

### Model formulation and assumptions

**Fig 1:**
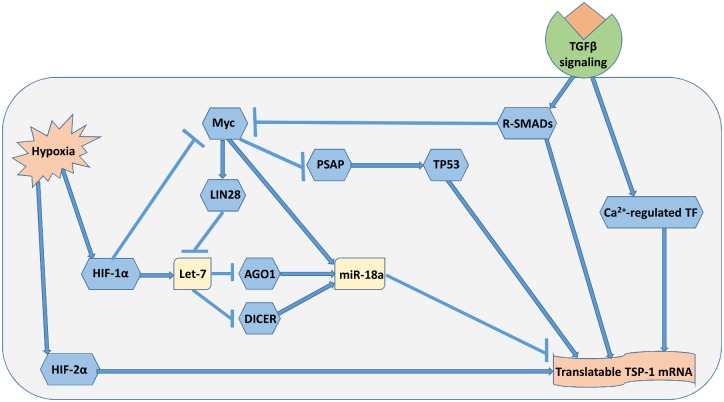
Induction of TSP-1 via multiple mechanisms by hypoxia and TGFβ signaling. An increase in the abundance of translatable TSP-1 mRNA in hypoxia results from the regulation by different pathways. Arrow symbol denotes activation, ⊣ symbol denotes repression. MicroRNAs that target TSP-1 (e.g. miR-18a) are less abundant in hypoxic conditions, together with the activation of different TSP-1 promoters, lead to an increase in intracellular TSP-1 production.

**Fig 2:**
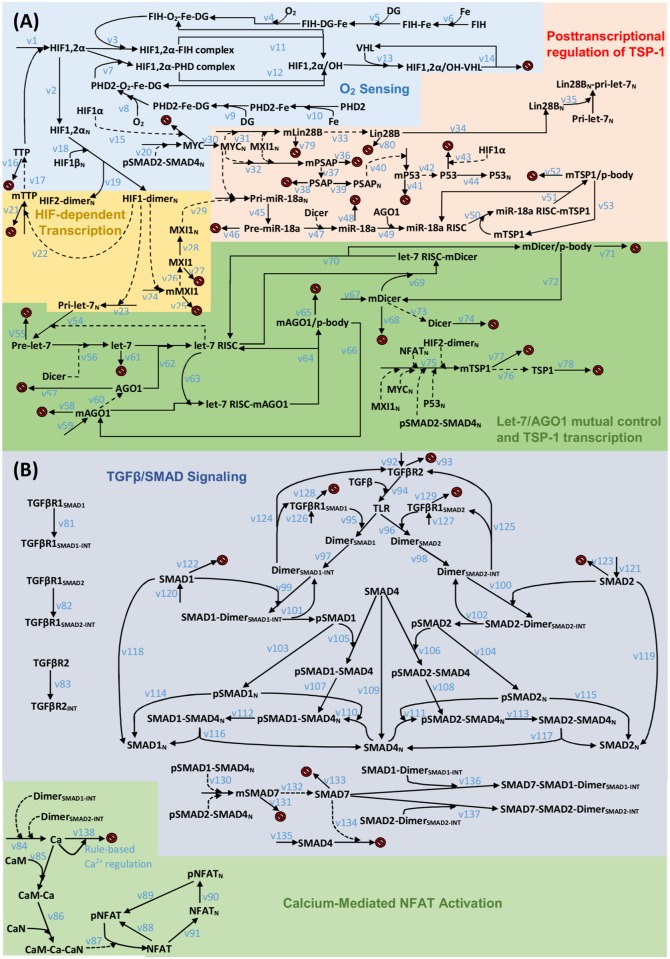
Reaction diagram of TSP-1 regulation by hypoxia and TGFβ signaling. (A) HIF stabilization in hypoxia, induction of let-7 and regulation of TSP-1 mRNA by miR-18a. Transcription of TSP-1 gene is modulated by different factors. (B) TGFβ signaling and calcium-mediated activation of NFATc1. Species whose names end with an N subscript are located inside the nucleus; reactions that point to red signs indicate degradation. The symbols v# in the two subparts (A and B) refer to the chemical reactions listed in S1 Table.

The computational model presented in this study describes intracellular synthesis of TSP-1 in ECs under the control of multiple signaling axes (Fig 1). The detailed reaction networks are divided into two subparts, (A) intracellular TSP-1 regulation and (B) TGFβ activation of TSP-1, and the diagrams are shown in Fig 2A and 2B. TGFβ pathways have been reported to play profound roles in cancer and cardiovascular diseases; in both situations, the anti-angiogenic effect of the downstream target TSP-1 can be harnessed therapeutically [33–35]. Established models of TGFβ signaling are available in the literature and they cover a wide range of biological details including TGFβ receptors, SMADs and phosphatases in different cellular compartments [36–38]. Due to model complexity concerns, the TGFβ signaling pathway in our model is an adapted version of the work by Nicklas and Saiz, where they included receptor binding, trafficking, SMAD activation, shuttling and feedback [39]. In addition, we implemented a different module of SMAD7-induced feedback and added the detail of SMAD7-mediated SMAD4 degradation, while SMAD4 is a co-SMAD that binds receptor-regulated SMADs (R-SMADs) [40, 41]. Also, a component of the TGFβ-induced calcium signaling network is included in our model with a few rule-based reactions dictating the rate of calcium influx and outflux upon TGFβ activation (see S1 Fig and S1 Table). Calcium binds and activates calmodulin and calcineurin sequentially, and activated calcineurin rapidly dephosphorylates the inactive NFATc1 in the cytoplasm [42]. Dephosphorylated NFATc1 is then shuttled into the nucleus and it promotes TSP-1 transcription; NFATc1 may be phosphorylated again and it aggregates in the cytoplasm in its inactive form [28, 43]. The current model does not include the potential contribution of calcium to the TGFβ-dependent SMAD activations or the direct binding between calcium and TSP-1 [44, 45]. It is important to note that although the signaling events downstream of TSP-1/receptor ligation are not covered in this model, they are reported to be the major effectors of the anti-angiogenic and pro-inflammatory properties of TSP-1 by regulating various molecules including but not limited to reactive oxygen species, Myc, nitric oxide, cyclic guanosine monophosphate (cGMP) and cyclic adenosine monophosphate (cAMP) [26, 46–50].

The intracellular regulation of TSP-1 synthesis in the model is primarily driven by hypoxia, an important stress signal in tumors and in PAD, through multiple signaling cascades that connect to the HIFs. The oxygen sensing module is similar to the one described by Zhao and Popel, in which they included hydroxylation of HIF mediated by iron, 2-oxoglutarate, PHD (prolyl hydroxylase domain-containing protein) and FIH (factor inhibiting HIF) as key species and processes during HIF stabilization [22]. The mechanism of HIF-2α stabilization in hypoxia is similar to that of HIF-1α, but the HIF-2 dimer, compared to HIF-1 dimer, is suggested to be a more dominant activator of TSP-1 transcription [14, 51, 52]. Hypoxia-driven induction of HIF-1α promotes the transcription of let-7, a hypoxia-responsive miR (HRM), while the ability of HIF-2α to induce HRMs is similar to that of HIF-1α and thus is not included considering model complexity reduction [23]. Myc and tumor protein 53 (p53), whose expressions are shown to be affected by hypoxia, have been identified as upstream regulators of TSP-1 with opposing impacts [53, 54]. Accumulated HIF-1α potently regulates the expression of Myc by directly promoting its degradation and inducing the MXI-1 (MAX interactor 1) protein which downregulates the transcriptional activity of Myc [25]. We assumed that MXI-1 exerts opposite transcriptional activity with respect to Myc on all of its target genes in the model. Myc is considered a weak transcriptional repressor of TSP-1 [18]. Besides this direct interaction, the downregulation of Myc can significantly contribute to TSP-1 induction by upregulating Prosaposin (PSAP) which leads to increased expression of p53, a positive promoter of TSP-1 transcription, and by downregulating the microRNAs that target TSP-1 mRNA [55, 56]. HIF-1α accumulated in hypoxia represses the proteasomal degradation of p53 [57, 58].

The microRNAs described in the model include let-7 and miR-18a. MicroRNA let-7 plays a master role in the regulation of AGO1 and Dicer which together strongly limit the global microRNA biogenesis in hypoxia [23, 59, 60]. Myc also negatively regulates the abundance of let-7 by inducing the Lin28B (Lin-28 Homolog B) protein which impairs the processing of let-7 primary transcripts in the nucleus [61, 62]. The other microRNA, miR-18a, is included in our model to represent the few confirmed TSP-1-targeting miRs and it is reported to be a direct repressor of TSP-1 mRNA in ECs, colonocytes and cardiomyocytes [19, 63, 64]. It is found that expression of miR-18a strongly depends on the transcriptional activity of Myc, which might be part of an indirect mechanism in the Myc-mediated TSP-1 repression [65]. All the biochemical reactions involving the mechanistic activities of miRs follow the detailed miR biogenesis/targeting mechanisms modeled previously by Zhao and Popel [22]. The two model subparts converge on the gene transcription of TSP-1, which depends on the activities of multiple transcription factors including HIF-2α, Myc, nuclear phosphorylated SMAD2-SMAD4 complex, nuclear active NFATc1, and p53 in an multiplicative manner [14, 18, 27, 28, 55]. One potential connection between the intracellular TSP-1 regulation and the TGFβ activation of TSP-1 is through the activation of the TGFβ pathway which represses the activity of Myc [66]. In the model, the synthesis of Myc is regulated by the signal downstream of TGFβ activation, which is simplified as the nuclear phosphorylated SMAD2-SMAD4 complexes [67, 68]. Another model assumption is that only the proteins/miRs located in the cytoplasm can undergo degradation, and the phosphorylation of SMADs takes place only in the cytoplasm. In microarray data that profile mRNA expression in C57BL/6 mouse with or without experimental hindlimb ischemia, TSP-1 and NFAT are among the top 5% most upregulated genes and MDM2 (Mouse double minute 2 homolog, E3 ubiquitin-protein ligase), which promotes p53 degradation, is in the top 5% most downregulated genes in the ischemic group compared to the non-ischemic group; in addition, MYCT1 (Myc target protein 1), whose transcription is directly influenced by Myc availability, is also modestly downregulated in the ischemic group [69, 70]. This evidence supports our model formulation hypothesis that NFAT, Myc and p53 are potential key players in the intracellular regulation of TSP-1. The model contains over 100 species and nearly 200 parameters (see S1 Table and Methods for details); except the small portion of parameters whose values have been measured and calculated in previous studies, the rest of the parameters are estimated by conducting model optimization and validation against literature experimental data (a total of 41 time-course expression trajectories of pathway signature molecules including over 200 data points).

### Model optimization and validation

**Fig 3:**
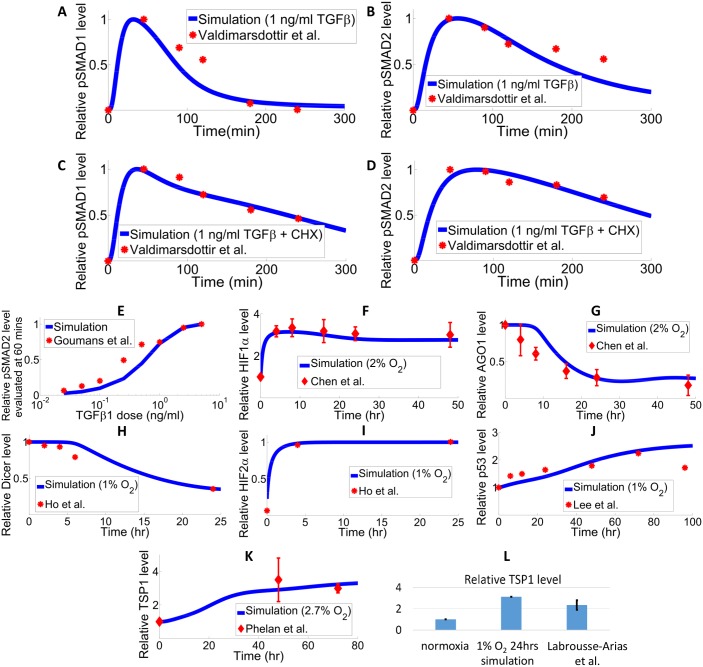
Model optimization against experimental data in ECs. (A-D) Experimental measurement and model simulation of phosphorylated SMAD1 and SMAD2 protein in response to 1 ng/ml TGFβ treatment in BAECs: (A) normalized phosphorylated SMAD1 protein level without CHX treatment, (B) normalized phosphorylated SMAD2 protein level without CHX treatment, (C) normalized phosphorylated SMAD1 protein level with CHX treatment, (D) normalized phosphorylated SMAD2 protein level with CHX treatment. The time-course experimental data from Valdimarsdottir et al. and simulation results are both normalized with respect to the corresponding peak value [71]. (E) Experimental data from Goumans et al. and model-generated dose response curve of total phosphorylated SMAD2 protein measured at 60 minutes after TGFβ treatment. X-axis is in log scale. Values are normalized against the maximum of each dataset [72]. (F) HIF-1α protein stabilization and (G) AGO1 protein downregulation are observed in HUVECs in hypoxia (2% O_2_) by Chen et al [23]. (H-I) Dicer protein is downregulated by hypoxia (1% O_2_) in HUVECs; hypoxia results in accumulation of HIF-2α protein in human dermal microvascular ECs from Ho et al [59]. (J) P53 protein expression is induced in hypoxic conditions (1% O_2_) in HUVECs; experimental data from Lee et al [73]. (K) TSP-1 protein expression is increased in hypoxia in HUVECs (2.7% O_2_); data from Phelan et al [16]. (L) TSP-1 protein expression is induced in human pulmonary aortic endothelial cells in response to 24 hours of hypoxia; data from Labrousse-Arias et al [14]. (A-L) Results (data-point values and simulations) are normalized against the maximum (or the normoxic baseline values) in each dataset. Quantification of experimental data from the literature is done in ImageJ following standard protocols.

Model parameters are optimized as described in the Methods Section (see S1 Table) and model simulations are compared with experimental data obtained by different research groups. Valdimarsdottir et al. quantified the phosphorylated SMAD1 and SMAD2 in bovine aortic endothelial cells (BAECs), pretreated with and without the protein synthesis inhibitor cycloheximide (CHX), in response to 1 ng/ml TGFβ (4e-5 μM) [39, 71]. In the simulation, the protein synthesis rates of all species are set to zero to mimic the effect of CHX. Fig 3A–3D compare the model simulation with experimental data, and the results imply that CHX treatment prolongs the plateaus of phosphorylated SMAD1 and SMAD2. Fig 3E compares the model-generated dose response curve of total phosphorylated SMAD2 with data obtained in BAECs [72]. Fig 3F–3L compare the model simulations of time-course protein expressions of various species including HIF-1α, HIF-2α, AGO1, Dicer, p53 and TSP-1 with corresponding experimental data obtained in human ECs [14, 16, 23, 59, 73]. Both the experimental data and our model simulations show that HIF-1α, HIF-2α, p53 and TSP-1 protein expressions are induced in hypoxia while AGO1 and Dicer protein levels are downregulated. The simulated calcium and NFAT dynamics are compared to HUVEC (human umbilical vein endothelial cell) data in S1 Fig in which the simulated overall trend of NFAT activation following a single calcium transient mimics the experimental data [74]. To show that the basic EC model can be further modified to explain fibroblast data as a proof-of-concept analysis, additional model calibration using a different set of parameters optimized against experimental data obtained from fibroblasts are shown in S2 Fig.

**Fig 4:**
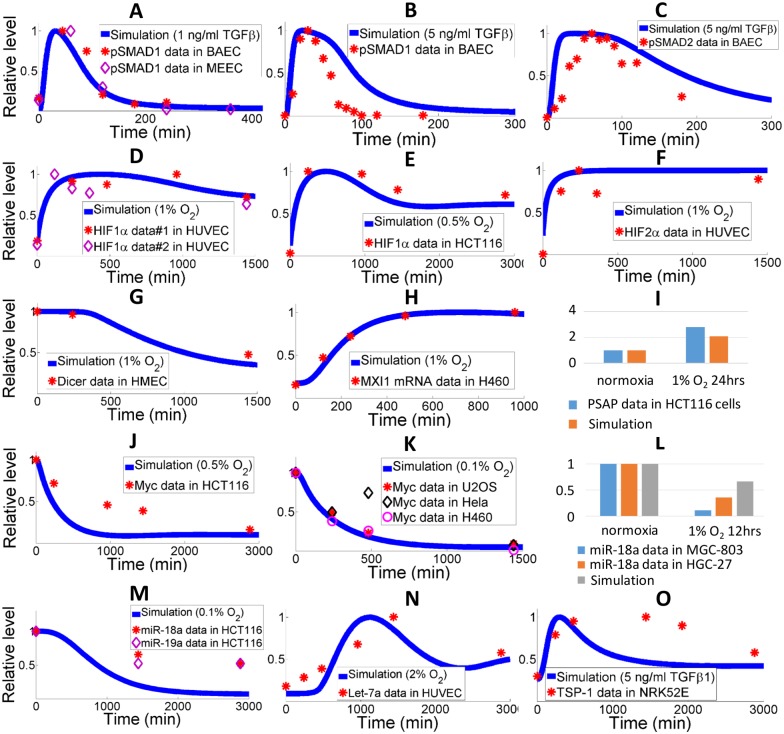
Qualitative model validation against experimental data in different cell types. (A-C) Experimental data (symbols) of total phosphorylated SMAD1 and SMAD2 protein in BAECs and mouse embryonic ECs when stimulated by different amount of TGFβ [71, 72]. Model simulations are presented by solid curves. (D-F) HIF1/2 protein expression data (symbols) measured in HUVECs and HCT116 colon carcinoma cells at different oxygen tensions [59, 75, 76]. Model simulations presented by solid curves show rapid induction of HIFs in hypoxia. (G) Dicer protein level (symbols) is decreased in hypoxia (1% O2) and measured in human dermal microvascular ECs [59]. Model simulation is presented by the solid curve. (H-I) Data on MXI-1 mRNA in H460 lung cancer cells and PSAP protein in HCT116 colon carcinoma cells indicate upregulation of both molecules in hypoxia [77, 78]. Model simulations confirm the trend. (J-K) Data on Myc protein in hypoxic conditions (symbols) in HCT116 cells, H460 cells, U2OS osteosarcoma cells and Hela cells [75, 77]. The downregulation of Myc is also captured by model simulations (solid curves). (L-M) Levels of miR-18a quantified in HCT116 cells, MGC-803 and HGC-27 gastric carcinoma cells [21, 79]. Model simulation of miR-18a downregulation in hypoxia agrees with experimental findings. The expression of miR-19a, another miR that potentially targets TSP-1, is also downregulated in hypoxia [19]. (N) Let-7a, together with other members of the let-7 miR family, belongs to the HRM group, and its induction is captured by model simulation (solid curve) and supported by experimental data (symbols) in HUVECs [23]. (O) Experimental data (symbols) in NRK52E rat kidney cells indicate that TSP-1 protein expression is stimulated by TGFβ signals [27]. Model simulation is shown by the solid curve. (A-O) Experimental data-point values and simulation curves are all normalized with respect to the peaks (or normoxic values in the bar-graphs). Literature data are quantified by ImageJ following standard protocols.

Given the novelty and the complexity of the model, validation is carried out in a way that the model simulations should reach qualitative agreements with uncalibrated experimental data obtained from a variety of different cell types (ECs, cancer cell lines, etc.), in order to partially resolve the issue of model parameter uncertainties. We have gathered additional experimental data from literature on the expression profiles of pathway signature molecules and the comparisons are shown in Fig 4A–4O. Without further calibration against these data, our model that runs with the parameter set obtained from the optimization process discussed above produces simulations that are able to match the trends and relative expression changes of those key molecules with satisfying accuracy. The “test dataset” shown in Fig 4 and the results in S2 Fig together suggest that the dynamics of key molecules in our model are qualitatively consistent in ECs, fibroblasts and certain cancer cell lines. This proof-of-concept step serves as a concrete theoretical basis for future experimental validations of our experiment-based computational model.

### Dose dependency of TSP-1 and SMAD7-mediated feedback

**Fig 5:**
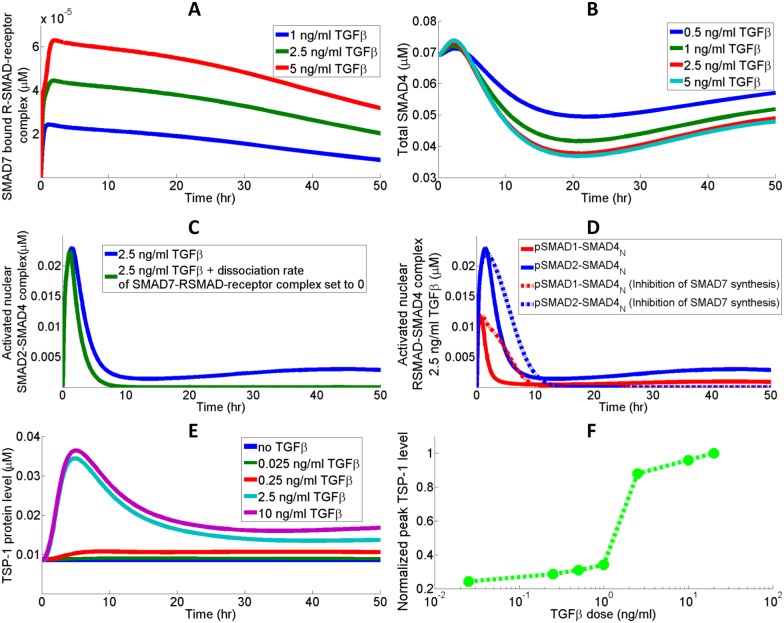
Dynamics of SMADs and TSP-1 protein synthesis. (A) SMAD7, whose expression is induced by TGFβ activation, binds the RSMAD-receptor complex and prevents the phosphorylation of the RSMAD in the complex. (B) SMAD4 abundance is negatively regulated by SMAD7 since accumulated SMAD7 promotes SMAD4 degradation. (C) RSMAD-receptor complex sequestered by SMAD7 upon TGFβ activation is released when SMAD7 expression decreases, giving rise to the tail expression of activated RSMAD-SMAD4. (D) Inhibition of SMAD7 protein synthesis removes the tail expression but significantly prolongs the RSMAD-SMAD4 activation signals. (E) TSP-1 protein synthesis stimulated by different doses of TGFβ and (F) the corresponding dose response curve produced by the model. The peaks of total intracellular TSP-1 levels are normalized with respect to the maximum peak observed in the simulation of 20 ng/ml TGFβ stimulation. X-axis is in log scale.

The SMAD proteins are the major effectors downstream of TGFβ. The R-SMADs, which typically refer to SMAD1/5 and SMAD2/3, are represented by SMAD1 and SMAD2 in the model [41, 80, 81]. SMAD7 induction follows the activation of the TGFβ pathway, and it associates with the R-SMAD-receptor complex to prevent phosphorylation of these R-SMADs by internalized receptors (Fig 5A). SMAD7 induction leads to a downregulation of SMAD4 in the cell by promoting its degradation (Fig 5B) [40]. In response to the rapid build-up of SMAD7 resulting from TGFβ receptor ligation, total SMAD4 level experiences an initial decay followed by a phase of slow restoration as TGFβ signal diminishes (Fig 5B). The tail expression of phosphorylated RSMAD-SMAD4 after 20 hrs in Fig 5C and 5D is an outcome of the reduced inhibitory effect of SMAD7: when SMAD7 expression is reduced, some of the sequestered R-SMAD-receptor complex is freed and is able to re-initiate the activation signaling cascade. SMAD7 primarily exerts its inhibitory effect during the peak of TGFβ activation, so a block of its synthesis should enhance R-SMAD phosphorylation and prolong the R-SMAD activation signal following the peak (Fig 5D). The tail expression of nuclear phosphorylated RSMAD-SMAD4 is not present when SMAD7 synthesis is inhibited due to the reduced binding between SMAD7 and R-SMAD-receptor complex. Given the dependency of TSP-1 promoter (SMAD2, NFATc1) activation on TGFβ-mediated signaling events, the TSP-1 synthesis curves produced by the model in response to different doses of TGFβ have similar trends compared to the time-course activation of R-SMADs, and the peak TSP-1 levels evaluated at around 10 hrs are shown to be dose dependent (Fig 5E and 5F). Model simulations suggest that TSP-1 protein synthesis is significantly elevated compared to the baseline level at TGFβ doses greater than 1 ng/ml, which supports the experimental findings by Nakagawa et al [27].

### Hypoxia mediates TSP-1 synthesis by promoting its transcription and inhibiting its mRNA repression

**Fig 6:**
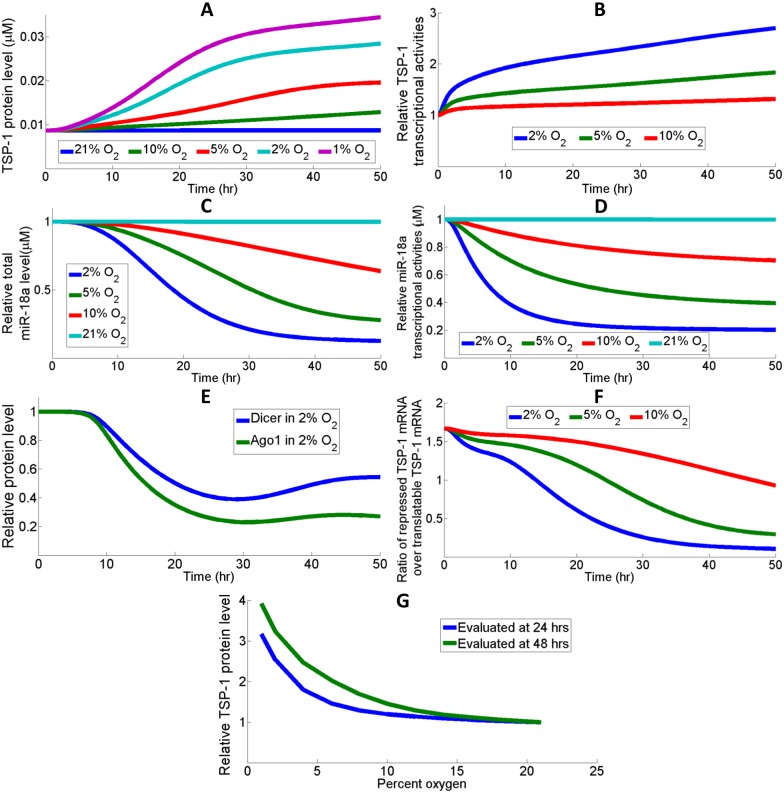
Low oxygen induces TSP-1 expression by different mechanisms. (A) TSP-1 protein expression is increased along with decreased oxygen availability. (B) Hypoxia increases the transcriptional activity of TSP-1 gene through the induction of its promoters. (C-D) miR-18a, a TSP-1 targeting miR, is downregulated in hypoxia due to the downregulation of its promoter Myc, and (E) miR processing molecules AGO1 and Dicer. (F) Hypoxia de-suppresses the TSP-1 mRNA that was originally under miR-mediated repression, which contributes to the increase of total translatable TSP-1 mRNA. The ratio of repressed TSP-1 mRNA over free mRNA decreases more than tenfold in the condition of 2% oxygen. (G) Model prediction of a nonlinear behavior in TSP-1 synthesis with respect to oxygen tension. This relationship may be partially contributed by the switch-like nature of cellular oxygen sensing [22, 82, 84]. (B-G) Results are normalized with respect to the normoxic baseline values.

The dynamic cooperation between transcriptional and posttranscriptional regulation of TSP-1 may be critical in its induction in response to hypoxia. Fig 6A shows the different TSP-1 induction profiles under different oxygen tensions. The increase in transcriptional activity gives rise to the increase in TSP-1 mRNA available for translation in hypoxia (Fig 6B). Another factor that the model hypothesizes to have contributed to the high expression of TSP-1 in hypoxia is the repression of microRNAs (e.g. miR-18a) that target TSP-1 (S3A Fig) [19, 21]. According to the simulations, downregulation of miR-18a in hypoxia is associated with a decrease in the production rate of miR-18a primary transcript due to repressed Myc expressions (S3B Fig) as well as decreased quantities of miR processing molecules, Dicer and AGO1 (Fig 6C–6E). Less TSP-1 mRNA is under repression in hypoxia while more mRNA is ready for translation due to the increased transcription and decreased miR targeting (Fig 6F). Since the stabilization of HIF is highly nonlinear with respect to oxygen tension and HIF initiates TSP-1 activation by multiple mechanisms, we wonder if there is also a switch-like behavior in the synthesis of TSP-1 at different oxygen tensions [82]. Fig 6G shows the normalized TSP-1 protein level as a function of percent oxygen, and the model predicts a nonlinear relationship when TSP-1 level is measured at both 24 hours and 48 hours; the threshold for induction of TSP-1 is centered around 6–8% oxygen. Since the model is constructed based on *in vitro* data, it should be noted that physiological tissue oxygen tension *in vivo* is usually much lower than 21% oxygen (*in vitro* normoxia), and such a discrepancy may affect our model conclusions when compared with *in vivo* experimental observations [83].

### Therapeutic strategies targeting the TGFβ-HIF-miR-TSP1 axis in diseases

Research on TSP-1 has established its promising role as future therapeutics in cancer and vascular disorders [85–88]. Computational studies such as our model may help design experiments to select the best strategy to modulate TSP-1 expression in these pathological conditions by running *in silico* experiments and assessing the results. In the following two subsections, we investigate how different factors contribute to TSP-1 dysregulation in tumors and in PAD and compare the efficacy of different model-motivated therapeutics.

#### Tumor

**Fig 7:**
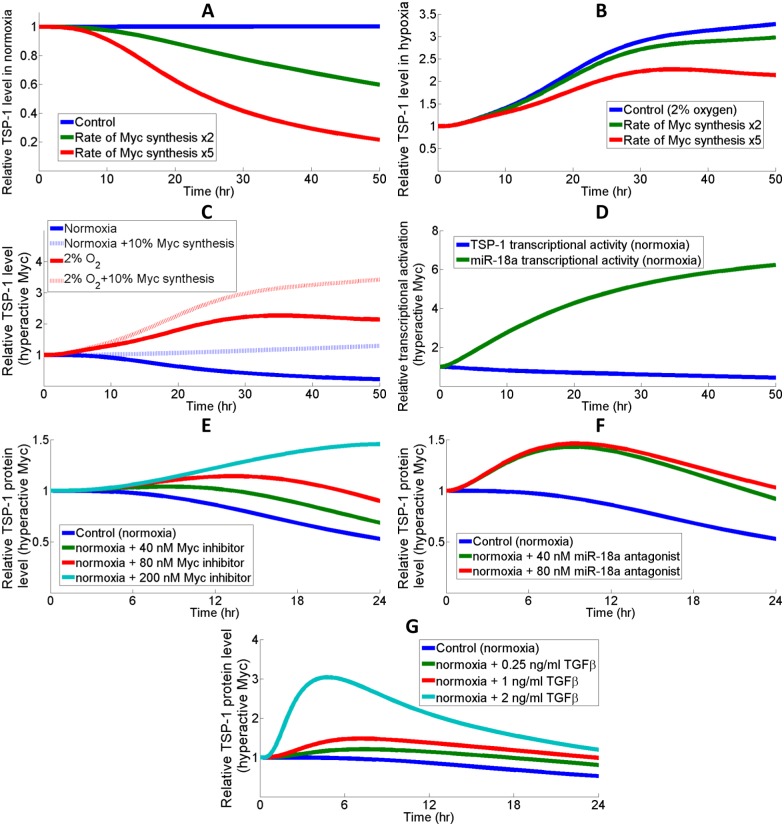
Different therapeutic interventions to restore TSP-1 level in Myc-induced tumors. (A) Hyperactivity of Myc is simulated by increasing its rate of production (baseline rate multiplied by 5), which results in a significant downregulation of TSP-1 protein level. Experimental evidence indicates that Myc expression could be 2–10 times higher in tumor samples compared with control samples [99–102]. (B) The effect of TSP-1 repression by Myc overexpression is less obvious in hypoxia. (C) In the cases of Myc hyperactivity, knockdown of Myc synthesis (protein synthesis rate reduced to 10% of the hyperactive value) leads to a more notable increase in TSP-1 protein abundance in normoxia compared to hypoxia. (D) Hyperactive Myc engages both the direct transcriptional and posttranscriptional pathways (miRs) to repress TSP-1 protein expression. (E) Simulating the application of a Myc inhibitor at different doses under the condition of Myc hyperactivity. (F) Simulating the application of a miR-18a antagonist at different doses under the condition of Myc hyperactivity. Simulation results suggest that miR antagonists reach a maximum efficacy at around 40 nM. (G) Direct transcriptional stimulation by different doses of TGFβ results in a significant early increase in TSP-1 protein expression under Myc hyperactivity compared to the other two strategies. (A-G) Results are normalized with respect to the normoxic steady-state values calculated with baseline Myc synthesis rate. (E-F) The simulations assume that Myc inhibitors potently bind and sequester cytoplasmic Myc with a Kd of 1 nM, and that miR-18a antagonists bind and sequester miR-18a RISC with a Kd of 1 nM [103].

Studies have confirmed that the enforced expression of TSP-1 in certain types of cancer, including lung and breast cancer, were associated with reduced tumor growth and metastasis [89–91]. Since Myc oncogene is often found overexpressed in tumors and given the connection between Myc and TSP-1, one potential mechanism contributing to the Myc-induced tumorigenesis is via the downregulation of TSP-1 [18, 92–94]. Therefore, enhancing TSP-1 expressions in these scenarios would provide anti-tumor benefits putatively by limiting angiogenesis and downregulating Myc [95]. The model suggests that in normoxia, Myc overexpression significantly downregulates the abundance of TSP-1 proteins (Fig 7A). However, in hypoxia, the contribution of Myc hyperactivity to TSP-1 downregulation is less significant and a simulated knockdown of Myc induces TSP-1 with a smaller fold increase (~1.5 folds at 48 hrs) compared to the substantial upregulation in normoxia (~5 folds at 48 hrs) (Fig 7B and 7C). This can be explained by the observation that hypoxia strongly downregulates Myc so the additional effect of reduced Myc synthesis on TSP-1 expression is less significant. In normoxia, hyperactive Myc does not repress TSP-1 transcription significantly, while it dramatically induces miR-18a production which results in increased TSP-1 mRNA repression (Fig 7D). Myc-induced miR-18a upregulation is an outcome of elevation in both transcriptional activation and AGO1 abundance, which helps to stabilize miRs and is controlled by the Myc-Lin28B-let7 axis (S4 Fig) [23, 61]. In order to reverse the downregulation of TSP-1 in simulations mimicking Myc-induced tumors, we test and assess several pathway-related therapeutic interventions including small molecule inhibitors of Myc, miR-18a antagonists and TGFβ treatments (Figs 7E–7G and S5) [96]. In a span of 24 hours, various doses of Myc inhibitors and miR-18a antagonists both elevated TSP-1 production by different amounts, while a rapid inhibition of Myc activity using a very high dose (200 nM) of Myc inhibitors successfully enhanced TSP-1 expression beyond normoxic steady-state level (simulation starting point) (Fig 7E and 7F). Forced TGFβ stimulation of TSP-1 may also be an effective strategy in the cases of Myc hyperactivity, since TGFβ signaling delays Myc synthesis (Figs 7G and S5D). The simulation results suggest that miR antagonists and TGFβ stimulations are more effective in terms of restoring TSP-1 expression in the early phase, while Myc inhibitors could give a higher TSP-1 expression in the long term (evaluated at 24 hrs). Although hypoxia suppresses Myc expression, the model predicts that hyperactivity of Myc due to gain-of-function mutations/deregulations still hinders the hypoxic induction of TSP-1 (Fig 7B), suggesting that cells produce insufficient anti-angiogenic factors (e.g. TSP-1) in these scenarios which fails to counteract the strong hypoxia-driven activation of pro-angiogenic factors (e.g. VEGF), and that this imbalance might be one possible reason that turns on the angiogenic switch [97, 98].

#### Peripheral arterial disease

**Fig 8:**
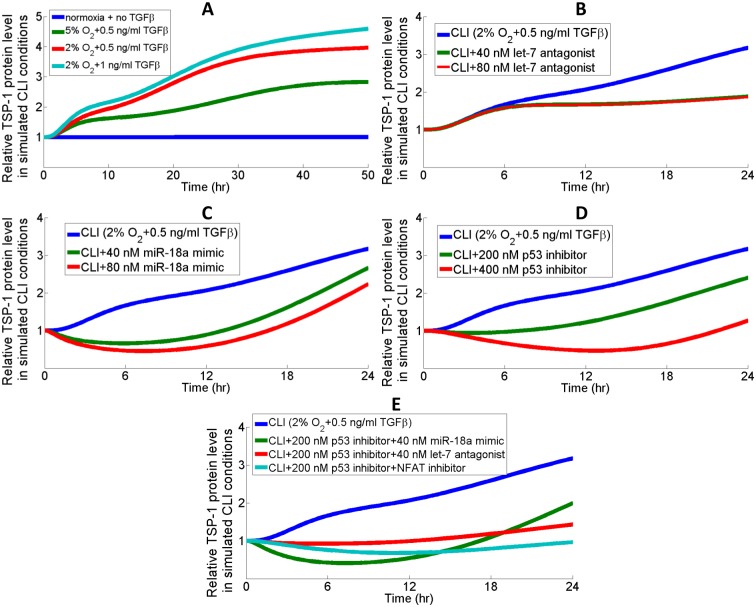
Different therapeutic interventions to reduce TSP-1 levels in PAD. (A) Hypoxia and TGFβ together contribute to a much higher intracellular TSP-1 protein expression in the simulated condition of CLI than the normoxic control condition (with no TGFβ). TSP-1 expression curves in response to different doses of (B) let-7 antagonists, (C) miR-18a mimics, and (D) p53 inhibitors. The effect of let-7 antagonist peaks at around 40 nM. (E) A combination of inhibiting both p53 production and NFAT activities achieve the most significant downregulation of TSP-1 in the simulated condition of CLI. In the simulations, the strength of NFAT inhibitor VIVIT is estimated from literature data to be a 70% decrease in the rate of calcineurin-mediated NFAT dephosphorylation when applied at micromolar doses [111]. (B-E) MiR-18a overexpression is simulated as an increase in the initial condition of precursor miR-18a; let-7 antagonists bind and sequester let-7 RISC with a Kd of 1 nM; small molecule inhibitor of p53 binds and sequesters cytoplasmic p53 with a Kd of 1 nM.

Research has suggested a detrimental role of TGFβ signaling in the progression of PAD by measuring the levels of TGFβ and its receptors in ischemic tissues and in peripheral blood collected from patients [104–106]. We are interested in the effect of TGFβ-induced TSP-1 synthesis and potential interventions to reduce TSP-1 in both normoxic and hypoxic conditions, since TSP-1 levels are found upregulated in PAD patients [13, 107]. In the most severe form of PAD, critical limb ischemia (CLI), arterial blood flow is restricted so severely that tissues are constantly suffering from hypoxia and ischemia [108]. Therefore, we simulated the condition of CLI as a combination of hypoxia and TGFβ which together contribute to elevated TSP-1 expressions (Fig 8A). Fig 8B–8D explore different potential treatment strategies during a 24-hr simulation timespan; under the simulated condition of CLI, inhibition of p53 is shown to be more effective in limiting TSP-1 production than antagonizing let-7 or overexpressing miR-18a (directly targets TSP-1 mRNA). Fig 8E compares time-course TSP-1 protein expressions in response to different combination therapies that this work proposed. A combination of both p53 and NFAT inhibition, designed to limit TSP-1 production by inhibiting both the hypoxia-induced and TGFβ-induced pathways, can most effectively reduce TSP-1 protein expression below the normoxic steady-state level (without TGFβ treatment) at the end of 24 hrs. NFAT inhibitors (e.g. VIVIT) specifically block the calcineurin-NFAT interaction and abolish the gene activation downstream of NFAT, which is assumed in the model to be activated upon TGFβ-induced calcium influx [109, 110].

### Model sensitivity analysis

**Fig 9:**
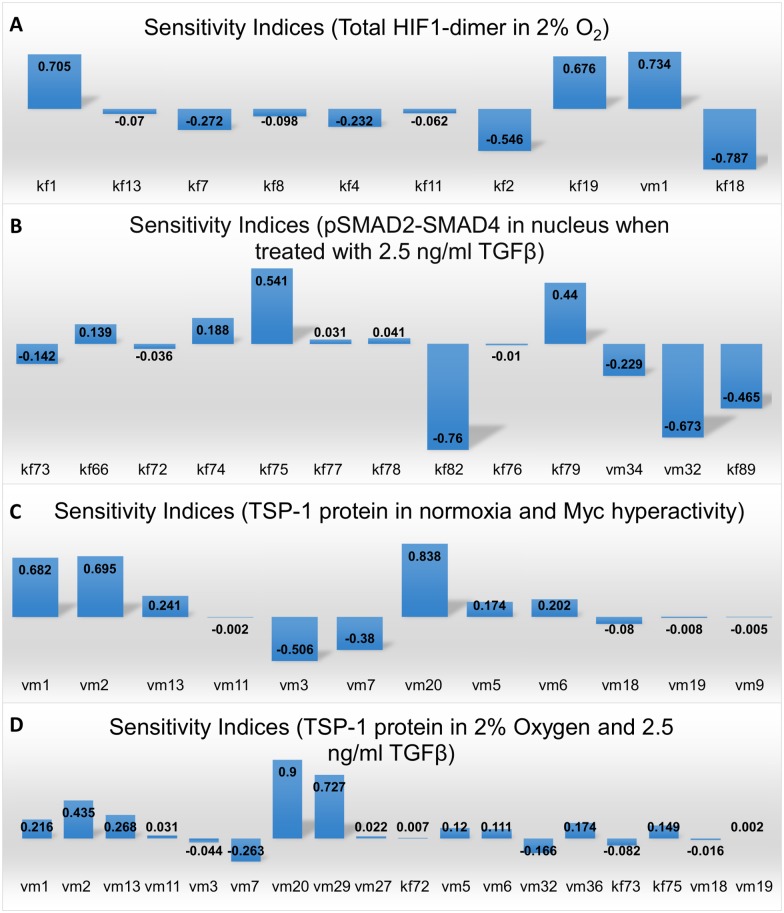
Global sensitivity analysis of model parameters. Global sensitivity analysis of parameters that control (A) the area under curve (AUC) of HIF-1 dimer in a span of 48 hours in 2% oxygen, (B) AUC of activated SMAD2-SMAD4 complex in nucleus in a span of 24 hours upon 2.5 ng/ml TGFβ stimulation, (C) AUC of TSP-1 in a span of 24 hours under the condition of normoxia plus Myc hyperactivity and (D) AUC of TSP-1 in a span of 24 hours upon 2.5 ng/ml TGFβ stimulation plus hypoxia (2% oxygen). (A-D) Rate descriptions—kf1: HIF-1α translocation into nucleus; kf2: HIF-1α binds FIH complex; kf4: oxygen binds FIH complex; kf7: HIF-1α binds PHD complex; kf8: oxygen binds PHD complex; kf11: HIF1α-OH-FIH dissociation; kf13: HIF1α-OH-PHD dissociation; kf18: TTP synthesis; kf19: HIF-1α binds HIF-1β; vm1: HIF-1α synthesis; vm2: HIF-2α synthesis; vm3: Myc synthesis; vm5: let-7 synthesis; vm6: MXI-1 synthesis; vm7: miR-18a synthesis; vm9: LIN28B synthesis; vm11: PSAP synthesis; vm13: p53 synthesis; vm18: AGO1 synthesis; vm19: Dicer synthesis; vm20: TSP-1 synthesis; kf66: TGFβR internalization; kf72: TGFβR degradation; kf73: TGFβ binds its receptor; kf74: receptor dimer binds R-SMAD; kf75: R-SMAD phosphorylation; kf76: R-SMAD translocation into nucleus; kf77: phosphorylated R-SMAD (pR-SMAD) binds SMAD4; kf78: pR-SMAD-SMAD4 complex translocation into nucleus; kf79: SMAD4 translocation into nucleus; kf82: pSMAD2-SMAD4 dephosphorylation; kf89: SMAD7 sequesters activated R-SMAD; vm27: TGFβ-mediated calcium influx; vm29: NFAT dephosphorylation; vm32: SMAD7 synthesis; vm34: SMAD4 degradation; vm36: SMAD4 synthesis.

We performed global sensitivity analysis using the techniques of Partial Rank Correlation Coefficient (PRCC, see Methods) under different simulated conditions to identify parameters that most significantly control the key species in the model [112]. S6 Fig displays the distribution of model parameters and the corresponding experimental measurements [113, 114]. Most of the parameter values after optimization are within one-two orders of magnitude compared to the experimental median values. Certain parameter values that deviate significantly from the experimental median are calculated based on literature data, such as the constitutive degradation rates of TGFβR (0.0278 min^-1^) in S6B Fig and the calmodulin concentration (5.9371 μM) in S6D Fig [36, 115]. From the sensitivity analysis, we observed that the HIF-1 dimer level, an indicator of HIF-mediated transcriptional activities in hypoxia, is negatively regulated by an increase in the affinity between HIF-1α and its two hydroxylases, FIH and PHD, which will subsequently promote HIF-1α degradation; as expected, increased binding between oxygen and FIH/PHD-DG-Fe complex (parameters kf4, kf8) speeds up the degradation of HIF (Fig 9A). Interestingly, although increased dimerization between HIF-1α and HIF-1β (parameter kf19) increases HIF-1 dimer levels, it downregulates the total HIF-1α protein level within the cell, presumably due to a higher synthesis of the HIF-destabilizing protein TTP (parameter kf18) as described by previous studies (Figs 9A and S7A) [116, 117]. Phosphorylation rate of cytoplasmic SMAD2 (parameter kf75) and SMAD4 shuttling rate (from cytoplasm to nucleus, parameter kf79) are the two most influential factors that positively regulate the levels of active, phosphorylated SMAD2-SMAD4 complex in nucleus, which represents the signaling strength of TGFβ pathways (Fig 9B). Besides the rates relating to SMAD7 feedback, increases in other factors such as the binding between TGFβ and its receptor (kf73) and the degradation of SMAD4 (vm34) are both correlated with less total activation of R-SMADs (Fig 9B). Sensitivity analysis of factors that control TSP-1 synthesis indicate that parameters relating to the abundance of transcription factors, including Myc, p53, HIFs, and NFAT are more influential (Fig 9C and 9D). Besides the strategies of TSP-1 or HIF gene therapies (parameters vm1, vm2, vm20), the model suggests that small molecule inhibitors against Myc and miR-18a can effectively restore and enhance TSP-1 protein expressions in tumorigenic conditions provoked by Myc overexpression (Fig 9C). In Fig 9D, manipulating the expression of miRs (let-7 or miR-18a alone) in simulated CLI conditions modulates TSP-1 production to a lesser extent compared to the approaches that directly target the transcription factors, and the results in Fig 8E also support the conclusion derived from the sensitivity analysis that targeting NFAT and p53 together may be a more efficient strategy. Additional results (S7 Fig) of model sensitivity in simulated conditions different from the ones presented in Fig 9 are consistent with the results discussed here. Given the potential interactions between different transcription factors (e.g. NFAT, HIF, p53, Myc) at the DNA level and the limited knowledge on how the influence of each individual promoter/inhibitor converge during TSP-1 transcription, the conclusions from the sensitivity analysis are biased by the simplification we made when translating the complex transcriptional activities into mathematical equations. The fact that our model assumed a multiplicative effect of different transcription factors is reflected by the results in Fig 9C and 9D that the TSP-1 protein level is relatively more sensitive to the parameters that control the abundance of its transcriptional promoters/repressors.

## Materials and Methods

### Formulation of reactions

We constructed the model based on ordinary differential equations (ODE) with a total of 109 species, 195 kinetic parameters and 138 reactions (Fig 2). Description of reactions, parameter values (S1 Table), and initial conditions for all species (S2 Table) are available in the appendices. The model allows translocation for certain species, especially the receptor and SMAD complexes, and distinguishes them by cellular locations–in cytoplasm, nucleus or endosome, since their functions are different in different cellular compartments. Transcriptional activation/repression and Dicer cleaving are modeled as Hill-type or Michaelis-Menten kinetics. Most interactions captured by the model are based on literature evidence. All data including reactions, rates, rules and initial conditions used in the model are compiled using MATLAB SimBiology toolbox (MathWorks, Natick, MA). Simulations are performed using the ode15s and sundials method, which are both ODE solvers provided in MATLAB. Since hypoxia is a focus of the study, the initial conditions of all species are their respective steady-state levels in simulations assuming normoxia (21% O_2_) and no TGFβ treatment. For miR treatments simulated by the model, overexpression of the miR mimic increases the initial condition of the corresponding precursor miR; miR silencing is described as the association of miR antagonist with miRISC to form a complex that cannot function. Our ODE-based computational model inherently considers time delays in biological events and is designed to simulate the average dynamical behavior of different biomolecules considering the stochasticity of cellular activities (binding, transcription, etc.), given the reasons stated in one of our previous works [22]. Although it is suggested that stochasticity plays a critical role in gene transcription, many signaling network studies that used deterministic approaches to model transcriptional events have been able to generate insightful results that are further validated by experiments [118–123]. Another reason why we did not use the stochastic approach to model transcription is that our study focuses primarily on the dynamic signal transduction and pathway cooperation within the network that together contribute to the induction/inhibition of TSP-1 protein expression in different circumstances, instead of the details in the transcription factor binding process at the DNA level. ImageJ software (NIH) is used to perform densitometry analysis according to the blot analysis protocol in order to obtain the experimental data showed in the model optimization and validation sections.

### Estimation of model parameters and initial conditions

Due to the limited literature on miR and TSP-1 modeling and the fact that this model is the first that describes the complex regulation responsible for TSP-1 synthesis under different physiological conditions, we paid considerable attention to parameter estimation and optimization during model construction. Many of the rate parameters and initial conditions used in the TGFβ signaling subpart are taken from the work by Nicklas and Saiz in which they calculated the values based on experimental measurements [39]. Parameters used in the component describing calcium-mediated NFAT activations are estimated and then optimized to reproduce the qualitative experimental behaviors of calcium and NFAT observed in ECs [74]. Intracellular concentrations of calcium are estimated based on data from [124]. For the initial conditions of miRs, we compared literature data and assumed that miR levels are on the order of 10^3^ to 10^4^ copies per cell in normoxia; the concentrations (in microMolar) used as initial conditions in the model are computed using 1 pL cell compartment volumes based on literature measurements [125–128]. Absolute levels of the different proteins in the model are estimated to be on the order of 10^4^ to 10^6^ copies per cell based on experimental measurements of several pathway-related proteins including Myc, p53 and calmodulin [115, 129, 130].

We estimated the decay rates of mRNA (1.2e-3 min^-1^), miRNA (1e-4 min^-1^), protein (2.5e-4 min^-1^), translation rate per mRNA (2.33 min^-1^), transcription/mRNA synthesis rate (1.92e-7 μM/min), and the levels of mRNA (2.8e-5 μM) and protein (0.08 μM) in normoxia so that the final values are within ±2 orders of magnitude compared to the median values (normalized by cell compartment volumes, and indicated in the brackets) reported by global quantification studies [22, 113, 114, 131, 132]. The rest of the parameters and initial conditions are estimated based on previous computational studies (summarized in S1 and S2 Tables) [22, 39, 84]. The volume concentrations of surface TGFβR are calculated by assuming that the receptors are distributed uniformly within a space of 1 pL given an estimated flat EC surface area of 1000 μm^2^ [133, 134]. We used the Levenberg-Marquardt algorithm within the *lsqnonlin* function in MATLAB for model optimization. Since the related time-course data in ECs are limited, the parameters are optimized by minimizing the sum of squared errors between normalized model simulations and experimental measurements (see Fig 3 in Results for details). The same protocol is repeated in the optimization of the model against the fibroblast dataset.

### Sensitivity analysis

Global sensitivity analysis is performed using the PRCC algorithm, a sampling-based method developed by Marino et al. to quantify uncertainty in the model. The outputs of interest in the sensitivity analysis are the time integrals of the signals computed in the form of AUC over certain durations, and a sample size of 1000 runs is chosen for each module of sensitivity analysis. The distribution of each parameter tested is within a two orders of magnitude range with a center at the parameter’s original value (e.g. x/10 to 10x). Details and examples of the PRCC algorithm can be found in [112].

## Discussion

In this study, a detailed mass-action based computational model of multiple signaling pathways connecting to TSP-1 regulation is presented. The comprehensiveness and trustworthiness of the model is supported by a careful analysis of literature during model formulation and extensive efforts of model training/validation against experimental data. This work is a continuation of a previous model presented by our group, while in that model VEGF is the major focus [22]. The scope of the current model is not limited to intracellular signaling since we included the module of TGFβ/receptor signaling as an important path of TSP-1 activation. TSP-1 is long known to be an activator of TGFβ, but the potential role of TGFβ on TSP-1 activation has not received much attention [135]. The model connects independent literature evidence and hypothesizes both a direct and indirect TSP-1 activation path initiated by TGFβ stimulation via SMADs and calcium regulation. This potential positive feedback loop that amplifies both TGFβ1 and TSP-1 expression might be an explanation to the paired high TGFβ1 and TSP-1 levels observed in certain pathological conditions [28, 136]. Although the regulatory roles of TSP-1 in tumor progression is highly cell-type specific, the undesirable anti-angiogenic effect resulting from high TSP-1 expression in PAD is a major interest to cardiologists and vascular biologists [107, 137, 138]. Our model proposed that TGFβ might be an underlying factor driving the high expressions of TSP-1 in PAD patients, given the experimental evidence of TGFβ1 elevation in ischemic tissues [106, 139]. It is interesting to note that hypoxia also upregulates TGFβ1 production in smooth muscle cells in addition to the direct transcriptional induction of TSP-1 via HIFs, suggesting another layer of crosstalk between the pathways that control TSP-1 expression [14]. The biology of hypoxia-induced TSP-1 seems contradictory to the need of angiogenesis when cells are exposed to insufficient oxygen, however, this phenomenon may be more likely an endogenous feedback control developed by the body to contain the angiogenesis driven by pro-angiogenic factors (e.g. VEGF) that are radically produced upon hypoxia [140].

The potential therapeutic interventions tested in this study to enhance TSP-1 production in simulated conditions of tumors are based on the assumption that these tumors are induced by Myc hyperactivity. Given the profound role of Myc in growth, proliferation, tumorigenesis and stem cells, the focus of our study, TSP-1, is only one of the many potential downstream targets of Myc that have correlations with tumor progression [92]. It is worth noting that Myc can induce miR-17/92 cluster which targets key proteins in TGFβ signal transduction and represses gene regulation downstream of TGFβ in multiple cancer cell lines, and that the pro-tumorigenic property of Myc overexpression is lost in TGFβ-deficient xenograft models of colorectal cancer; such evidence suggests that Myc may promote tumor growth primarily by repressing the anti-tumorigenic gene expression (including TSP-1) activated by TGFβ signaling, at least in the context of colorectal cancer [141, 142]. Moreover, the mutually inhibitory relationship between TSP-1 and Myc may further amplify the signal of one molecule and suppress the other in diseases [95]. VEGF is also shown to be a target activated by Myc [143, 144]. Although research has shown that TSP-1 overexpression can effectively reduce tumor metastasis, the position of TSP-1 in the entire network of cancer-related genes is relatively downstream, which might imply that targeting TSP-1 to attack tumor may be less efficacious than targeting the genes (e.g. Myc) that are more central in the network, since cancer is notorious for developing compensatory pathways to resist targeted therapies [145, 146]. The failure of TSP-1 analog (ABT-510) in phase II trials against metastatic cancer should not discourage the continuum of research that aims to explore the therapeutic potential of TSP-1, especially in cardiovascular diseases where its importance has emerged in recent years; on the other hand, multiple phase I studies that explore the targeting of CD47 in cancer, given its inhibitory effect on the immune response, are now under way [147–150]. Still, our simulations proposed that increased TSP-1 synthesis is a possible downstream effector of the tumor-suppressive property of TGFβ signaling, specifically in Myc-dependent tumors; however, the exact role of TGFβ in cancer is quite complex and controversial given its bipolar control of tumorigenesis [151–155].

Sensitivity analysis indicates that TSP-1 production stimulated by hypoxia and TGFβ is strongly influenced by the activity of several transcription factors, namely HIFs, p53 and NFAT. Although the model assumes that HIF-1 does not directly promote TSP-1 transcription, an increase in its abundance, as shown by the sensitivity analysis, has a notable influence on TSP-1 levels comparable to that of HIF-2, which directly activates TSP-1 [14]. The indirect activation of TSP-1 by HIF-1α might be undesired for gene therapies that use adenoviral HIF-1α to improve angiogenesis and limb perfusion in patients with ischemic vascular diseases [156]. This might also explain the finding that the angiogenic potency of adenoviral HIF-1α is significantly lower than that of adenoviral VEGF [157]. To date, the roles of p53 and NFAT, the two potential therapeutic targets identified by our simulations, in PAD are largely unknown. The limited evidence in the literature agrees with the model hypothesis that NFAT, with TSP-1 as one of its effector molecules, potently participates in the cellular response to hypoxia/ischemia: inhibition of NFAT is found to suppress atherosclerosis in diabetic mice, while a significant increase in NFAT expression is observed in ischemic rat brain [158, 159]. Tumor protein p53 is long known to be a critical factor in suppressing tumorigenesis and initiating apoptosis; its pro-apoptotic property might render it a promising target in PAD given multiple clinical observations of the increased level of apoptotic events in the serum and tissue of PAD and CAD (coronary artery disease) patients [160–163]. Still, the robustness and reliability of our model-based conclusions can be further enhanced by additional model training, calibration and validation when more experimental measurements (e.g. data of HIFs, miRs, AGOs, Myc, SMADs and TSP-1 expressions in different physiological condition/stimulation) become available in the near future.

The current model describes the dynamics of let-7 and miR-18a in intracellular regulation of TSP-1, but the model is set up in a way that incorporating additional miRs and their targets is feasible. Besides the miRs that target HIFs such as miR-155, many other miRs could be potential candidates to consider for future computational models of TSP-1 regulation [164]. In the current model simulations, we assume that inhibition of p53 is achieved by the binding of small molecule inhibitors (e.g. Cyclic Pifithrin-α), while p53 is reported to be a target of several miRs including miR-125b and miR-504 [165–167]. The p53 protein also regulates the expression of certain miRs; an example is miR-194, a p53-responsive miR which targets TSP-1 in colon cancer cell lines [56]. Liao et al. identified let-7g, one of the HRMs, as a factor that improves endothelial functions with targets including TSP-1 [168]. Future models could also consider alternative pathways relating to TSP-1 regulation that have implications in vascular disorders, such as the axis involving VEGF activation of NFAT in ECs [169]. The signaling pathway involving PI3K/AKT/PTEN is also shown to mediate TSP-1 expression in both cancer cells and ECs [170]. Including the VEGF signaling pathway as a part of intracellular TSP-1 regulation seems to be an exciting next step in the future development of our model, since most studies have focused on the regulatory effect of TSP-1 on VEGF but not the other direction. Besides hypoxia, many other factors relating to cancer and PAD including radiation, high glucose and aging have also been shown to affect TSP-1 expression [171–176]. So far, the model is mostly formulated and validated based on knowledge and data of pathways in ECs, which are shown in experimental studies to express TSP-1 at high levels and that the EC-secreted TSP-1 is critical in certain physiological processes [28, 177]. Given the fact that the amount of ECs is only a small percentage of all the cells in tissues of tumors or PAD, and other types of cells including smooth muscle cells, stromal fibroblasts and immune cells also secrete TSP-1, there is a need for further model validation in order to sustain and extend our model conclusion, at least qualitatively, to other cell types of interest [26]. We have already demonstrated the feasibility of this by conducting additional validation against experimental data from other cell types (e.g. cancer cells, fibroblasts etc.), but the related data in smooth and skeletal muscle cells are relatively scarce. A goal of this study is to raise carefully-formulated hypotheses that stimulate future research to produce additional experimental results that either corroborate or refute our predictions. In summary, our model is the first computational study that investigates the complex network of intracellular TSP-1 regulation mediated by hypoxia, microRNA-targeting and receptor signaling. It is an important complementary study to the active research that focuses on the interaction between TSP-1, VEGF and their receptors at the cell surface, and it also provides insights, from the perspective of intracellular control, into the search for therapeutic strategies that adjust TSP-1 activity in order to modulate angiogenesis in cancer and vascular diseases. Combined with additional modules of pharmacokinetic analysis, our computational model can help identify optimal treatment strategies and design appropriate dosing schemes that progressively reduce or enhance TSP-1 expression in patients depending on the specific indication.

## Supporting Information

S1 File Compiled file of all supporting information documents. (PDF)

S1 Table Reaction descriptions, reaction rates and kinetic parameters of TSP-1 model. (PDF)

S2 Table Differential equations and species initial conditions. (PDF)

S1 Fig Model of TGFβ-induced calcium regulation and downstream activation of NFAT in ECs. (PDF)

S2 Fig Additional model calibration against published fibroblast data using different parameter values. (PDF)

S3 Fig De-suppression of TSP-1 mRNA and downregulation of Myc in hypoxia. (PDF)

S4 Fig AGO1 upregulation following Myc overexpression. (PDF)

S5 Fig Testing different therapeutic strategies in hypoxia and hyperactive Myc conditions. (PDF)

S6 Fig Model parameter distributions. (PDF)

S7 Fig Additional sensitivity analysis. (PDF)
