# Supplementary material for: Transcriptional and Post-Transcriptional Regulation of Thrombospondin-1 Expression: A Computational Model
Source: PLoS Comput Biol. 2017 Jan 3;13(1):e1005272. doi: 10.1371/journal.pcbi.1005272 (PMC5207393; doi:10.1371/journal.pcbi.1005272)
Supplement: S1 File — (PDF) [file pcbi.1005272.s001.pdf]

S1_File

Supplementary information S1_File includes S1-S2 Tables, which contain the reactions, descriptions, parameters and initial conditions used in the model, and S1-S7_Figs which show additional related results.

S1_Table: Reaction descriptions, reaction rates, kinetic parameters of TSP-1 model

No.	Reaction description	Reaction Rates and Parameters (k#, v#, n#)	Reference
	**Intracellular TSP-1 regulation (Subpart A)**		
V1	Synthesis of HIFs	HIF1a: vm1*(1-[TTP]^n1/(kp1+[TTP]^n1)), vm1=0.03012 μM/min, n1=4, kp1=0.093 μM^4, HIF2a: vm2, vm2=0.096 μM/min	Vm1 and vm2 estimated from (1); fitted
V2	HIF shuttling into nucleus	HIF1a: kf1*[HIF1a] - kr1*[$HIF1a_N$], kf1=0.005 min^{-1}, kr1=0.018 min^{-1}, HIF2a: kf1*[HIF2a] – kr1*[$HIF2a_N$]	Fitted
V3	HIF binding with FIH complex	HIF1a: kf2*[HIF1a]*[FIH-O2-Fe-DG] – kr2*[HIF1a-FIH complex], kf2=0.13 $\mu M^{-1} min^{-1}$, kr2=1 min^{-1}, HIF2a: kf3*[HIF2a]*[FIH-O2-Fe-DG] – kr2*[HIF2a-FIH complex], kf3=1.3 $\mu M^{-1} min^{-1}$	Kf2 and kr2 estimated from (2); fitted
V4	Oxygen binding with FIH	kf4*[O2]*[FIH-DG-Fe] – kr4*[FIH-O2-Fe-DG], kf4=0.165 $\mu M^{-1} min^{-1}$, kr4=10.6 min^{-1}	Fitted; estimated from (2)
V5	2-OG binding with FIH	kf5*[FIH-Fe]*[DG] – kr5*[FIH-DG-Fe], kf5=0.23 $\mu M^{-1} min^{-1}$, kr5=7.4 min^{-1}	Estimated from (2)
V6	Iron binding with FIH	kf6*[Fe]*[FIH] – kr6*[FIH-Fe], kf6=4 $\mu M^{-1} min^{-1}$, kr6=10 min^{-1}	Fitted
V7	HIF binding with PHD complex	HIF1a: kf7*[PHD2-O2-Fe-DG]*[HIF1a] – kr7*[HIF1a-PHD complex], kf7=0.11 $\mu M^{-1} min^{-1}$, kr7=0.7 min^{-1}, HIF2a: kf7*[PHD2-O2-Fe-DG]*[HIF2a] – kr7*[HIF2a-PHD complex]	(2)
V8	Oxygen binding with PHD	kf8*[O2]*[PHD2-O2-Fe-DG] – kr8*[PHD2-O2-Fe-DG], kf8=0.043 $\mu M^{-1} min^{-1}$, kr8=10.8 min^{-1}	(2)

V9	2-OG binding with PHD	kf9*[DG]*[PHD2-Fe] – kr9*[PHD2-Fe-DG], kf9=0.18 $\mu M^{-1}min^{-1}$, kr9=10.8 min^{-1}	(2)
V10	Iron binding with PHD	kf10*[PHD2]*[Fe] – kr10*[PHD2-Fe], kf10=18 $\mu M^{-1}min^{-1}$, kr10=36 min^{-1}	(2)
V11	Hydroxylation of HIF-FIH complex	HIF1a: kf11*[HIF1a-FIH complex], kf11=34 min^{-1}, HIF2a: kf12*[HIF2a-FIH complex], kf12=0.34 min^{-1}	Fitted; estimated from (2)
V12	Hydroxylation of HIF-PHD complex	HIF1a: kf13*[HIF1a-PHD complex], kf13=0.44 min^{-1},<br>HIF2a: kf13*[HIF2a-PHD complex]	(2)
V13	VHL binding with hydroxylated HIF	HIF1a: kf14*[VHL]*[HIF1a-OH] – kr14*[HIF1a-OH-VHL], kf14=42 $\mu M^{-1}min^{-1}$, kr14=1.3 min^{-1}, HIF2a: kf14*[VHL]*[HIF2a-OH] – kr14*[HIF2a-OH-VHL]	(2)
V14	Degradation of HIF	HIF1a: kf15*[HIF1a-OH-VHL], kf15=1 $\mu M^{-1}min^{-1}$, HIF2a: kf15*[HIF2a-OH-VHL]	Fitted
V15	HIF1a promotes myc degradation	[MYC]*(kf16+vm2*([HIF1a]^n2/([HIF1a]^n2+kp2))), kf16=0.001 min^{-1}, vm2=0.05 min^{-1}, n2=2, kp2=3 μM^2	Kf16 estimated from (3); fitted
V16	Degradation of TTP protein	kf17*[TTP], kf17=0.002 min^{-1}	Estimated from (3)
V17	Protein translation of TTP	kf18*[mTTP], kf18=0.11 min^{-1}	Estimated from (3)
V18	Association between HIF1-α and HIF1-β	kf19*[HIF1b]*[HIF1a$_N$] - kr19*[HIF1-dimer$_N$], kf19=0.006 $\mu M^{-1}min^{-1}$, kr19=0.03 min^{-1}	Estimated from (4)
V19	Association between HIF2-α and HIF1-β	kf19*[HIF2a$_N$]*[HIF1b] - kr19*[HIF2-dimer$_N$]	
V20	SMAD inhibits Myc production	vm3*(1-[psmad2-smad4$_N$]/([psmad2-smad4$_N$]+kp3)), vm3=2.75e-5 μM/min, kp3=0.004 μM	Estimated from (1); fitted
V21	Degradation of TTP mRNA	kf20*[mTTP], kf20=0.004 min^{-1}	Estimated from (3, 5)
V22	HIF1 activates TTP production	vm4*([HIF1-dimer$_N$]^n4) /(kp4+([HIF1-dimer$_N$]^n4)), vm4=5e-5 μM/min, n4=2, kp4=9e-4 μM^2	Estimated from (6)
V23	HIF1 activates let-7	Vm5*([HIF1-dimer$_N$]^n5/(kp5+[HIF1-dimer$_N$]^n5)), vm5=1.0607e-4 μM/min, n5=3, kp5=5.181e-5 μM^3	Estimated from (6)
V24	HIF1 activates MXI-1	Vm6*[HIF1-dimer$_N$]^n6/([HIF1-dimer$_N$]^n6+kp6), vm6=6e-6 μM/min, n6=2, kp6=6.25e-4 μM^2	Estimated from (6)

V25	Degradation of MXI-1 mRNA	Kf21*[mMXI1], kf21=0.009 min^{-1}	Estimated from (3, 5)
V26	Protein translation of MXI-1	Kf22*[mMXI1], kf22=1 min^{-1}	Estimated from (3)
V27	Degradation of MXI-1 protein	Kf23*[MXI1], kf23=0.004 min^{-1}	Estimated from (3)
V28	MXI-1 shuttling into the nucleus	Kf24*[MXI1] – kr24*[$MXI1_N$], kf24=0.01 min^{-1}, kr24=0.004 min^{-1}	Fitted
V29	Transcription of miR-18a	Vm7*(vm8+[MYC]^n7/([MYC]^n7+kp7)) *(1-[$MXI1_N$]^n8/([$MXI1_N$]^n8+kp8)),vm7=6.8708e-5 µM/min,vm8=0.02,n7=2, kp7=0.01211 μM^2, n8=2, kp8=0.2 μM^2	Estimated from (6)
V30	Myc shuttling into the nucleus	Kf25*[MYC] – kr25*[MYC_N], kf25=0.01 min^{-1}, kr25=0.005 min^{-1}	Fitted
V31	Transcription of Lin28B	Vm9*([MYC_N]^n9)/(kp9+[MYC_N]^n9) *([$MXI1_N$]/([$MXI1_N$]+kp10)), vm9=4.354e-6 µM/min, n9=2, kp9=0.0151 μM^2, kp10=0.05 µM	Estimated from (6)
V32	Transcription of PSAP	Vm11* (1-[MYC_N]^n11/([MYC_N]^n11+kp11)) *([$MXI1_N$]/([$MXI1_N$]+kp12)), vm11=3.688e-7 µM/min, n11=2, kp11=6.054e-4 μM^2, kp12=0.06 µM	Estimated from (6)
V33	Protein translation of Lin28B	Kf26*[mLin28B], kf26=0.3 min^{-1}	Estimated from (3)
V34	Lin28B shuttling into the nucleus	Kf27*[Lin28B] – kr27*[$Lin28B_N$], kf27=0.03 min^{-1}, kr27=0.003 min^{-1}	Fitted
V35	Lin28B sequesters pri-let-7	Kf28*[$Lin28B_N$]*[pri-let-7_N] – kr28*[$Lin28B_N$-pri-let-7_N], kf28=4000 $\mu M^{-1} min^{-1}$, kr28=min^{-1}	Estimated from (7)
V36	Degradation of PSAP mRNA	Kf29*[mPSAP], kf29=0.002 min^{-1}	Estimated from (3, 5)
V37	Protein translation of PSAP	Kf30*[mPSAP], kf30=3 min^{-1}	Estimated from (3)
V38	Degradation of PSAP protein	Kf31*[PSAP], kf31=0.003 min^{-1}	Estimated from (3)
V39	PSAP shuttling into the nucleus	Kf32*[PSAP] – kr32*[$PSAP_N$], kf32=0.05 min^{-1}, kr32=0.02 min^{-1}	Fitted
V40	PSAP activates P53	Vm13*([$PSAP_N$]/([$PSAP_N$]+kp13)), vm13=4.402e-7 µM/min, kp13=0.15 µM	Estimated from (6)
V41	Degradation of p53 mRNA	Kf33*[mP53], kf33=0.0024^{-1}	Estimated from (3, 5)

V42	Protein translation of p53	Kf34*[mP53], kf34=4 min^{-1}	Estimated from (3)
V43	HIF1a prevents p53 degradation	Vm14*[P53]*(1-[HIF1a] /([HIF1a] +kp14)), vm14=0.00198 min^{-1}, kp14=2 μM	Estimated from (3); fitted
V44	P53 shuttling in the nucleus	Kf35*[P53] – kr35*[$P53_N$], kf35=0.08 min^{-1}, kr35=0.2 min^{-1}	Fitted
V45	Cleavage of pri-miR-18a	Kf36*[pri-miR-$18a_N$], kf36=0.007 min^{-1}	Fitted
V46	Degradation of pre-miR-18a	Kf37*[pre-miR-18a], kf37=0.006 min^{-1}	Fitted
V47	Cleavage of pre-miR-18a	Vm15*[Dicer]*([pre-mir-18a]/([pre-mir-18a]+kp15)), vm15=0.04 min^{-1}, kp15=0.05 μM	Fitted
V48	Degradation of miR-18a	Kf38*[miR-18a], kf38=0.003 min^{-1}	Estimated from (8)
V49	miR-18a binding AGO1	Kf39*[AGO1]*[miR-18a] – kr39*[miR-18a RISC], kf39=1.84 $\mu M^{-1} min^{-1}$, kr39=1.098 min^{-1}	Estimated from (7, 9)
V50	miR-18a RISC binding TSP-1 mRNA	Kf40*[miR-18a RISC]*[mTSP1] – kr40*[miR-18a RISC-mTSP1], kf40=2 $\mu M^{-1} min^{-1}$, kr40=0.23 min^{-1}	Fitted
V51	TSP-1 mRNA to p-body	Kf41*[miR-18a RISC-mTSP1], kf41=0.6 min^{-1}	Estimated from (10)
V52	TSP-1 mRNA degradation in the p-body	Kf42*[mTSP1/p-body], kf42=5e-4 min^{-1}	Fitted
V53	TSP-1 mRNA return to cytoplasm	Kf43*[mTSP1/p-body], kf43=0.002 min^{-1}	Estimated from (10)
V54	Cleavage of pri-let-7	[pri-let-7_N]*vm16*[Let-7 RISC]^n16/([Let-7 RISC]^n16+kp16), vm16=5 min^{-1}, n16=2, kp16=0.001215 μM^2	Fitted
V55	Degradation of pre-let-7	Kf44*[pre-let-7], kf44=0.01 min^{-1}	Fitted
V56	Cleavage of pre-let-7	Vm17*[Dicer]*[pre-let-7] /(kp17+[pre-let-7]), vm17=0.01 min^{-1}, kp17=0.03 μM	Fitted
V57	Protein degradation AGO1	Kf45*[AGO1], kf45=0.0021 min^{-1}	Estimated from (3)
V58	AGO1 mRNA degradation	Kf46*[mAGO1], kf46=1e-4 min^{-1}	Estimated from (3, 5)
V59	AGO1 mRNA synthesis	Vm18, vm18=6e-7 μM/min	Estimated from (6)
V60	Protein translation of AGO1	Kf47*[mAGO1], kf47=1.275 min^{-1}	Estimated from (3)

V61	Degradation of let-7	Kf48*[let-7], kf48=0.00803 min^{-1}	Estimated from (8)
V62	Let-7 binds AGO1	Kf49*[let-7]*[AGO1] – kr49*[let-7 RISC], kf49=1 $\mu M^{-1}min^{-1}$, kr49=0.07 min^{-1}	Estimated from (7, 9, 11)
V63	Let-7 RISC binds AGO1 mRNA	Kf50*[let-7 RISC]*[mAGO1]-kr50*[let-7 RISC-mAGO1], kf50=8 $\mu M^{-1}min^{-1}$, kr50=0.15 min^{-1}	Fitted
V64	AGO1 mRNA to p-body	Kf51*[let-7 RISC-mAGO1], kf51=0.3 min^{-1}	Estimated from (10)
V65	AGO1 mRNA degradation in p-body	Kf52*[mAGO1/p-body], kf52=2.63e-5 min^{-1}	Fitted
V66	AGO1 mRNA return to cytoplasm	Kf53*[mAGO1/p-body], kf53=0.001025 min^{-1}	Estimated from (10)
V67	Dicer mRNA synthesis	Vm19, vm19=2.4e-5 μM/min	Fitted
V68	Dicer mRNA degradation	Kf54*[mDicer], kf4=0.008 min^{-1}	Estimated from (3, 5)
V69	Let-7 RISC binds Dicer mRNA	Kf55*[let-7 RISC]*[mDicer]-kr55*[let-7 RISC-mDicer], kf55=6.9 $\mu M^{-1}min^{-1}$, kr55=0.09 min^{-1}	Fitted
V70	Dicer mRNA to p-body	Kf56*[let-7 RISC-mDicer], kf56=1.8 min^{-1}	Estimated from (10)
V71	Dicer mRNA degradation in p-body	Kf57*[mDicer/p-body], kf57=2e-5 min^{-1}	Fitted
V72	Dicer mRNA return to cytoplasm	Kf58*[mDicer/p-body], kf58=0.001 min^{-1}	Estimated from (10)
V73	Dicer protein translation	Kf59*[mDicer], kf59=0.5 min^{-1}	Estimated from (3)
V74	Dicer protein degradation	Kf60*[Dicer], kf60=0.0014 min^{-1}	Estimated from (3)
V75	TSP1 synthesis	Vm20*(vm21+[psmad2-smad4$_N$] /([psmad2-smad4$_N$]+kp21)) *([P53$_N$] /([P53$_N$] +kp22)) *(vm23+[NFAT$_N$] /([NFAT$_N$]+kp23)) *(([HIF2-dimer$_N$])/([HIF2-dimer$_N$]+kp24))* (1-([MYC$_N$])/([MYC$_N$]+kp25)) *([MXI1$_N$]/([MXI1$_N$] +kp26)), vm20=5.18e-5 μM/min, vm21=0.1, kp21=0.0899 μM, kp22=0.1 μM, vm23=0.001, kp23=0.00926 μM, kp24=0.015, kp25=0.11 μM, kp26=0.005 μM	Estimated from (6)
V76	TSP1 protein translation	Kf61*[mTSP1], kf61=1.84 min^{-1}	Estimated from (3)

V77	TSP1 mRNA degradation	Kf62*[mTSP1], kf62=0.003 min^{-1}	Estimated from (3, 5)
V78	TSP1 protein degradation	Kf63*[TSP1], kf63=0.005 min^{-1}	Estimated from (3)
V79	Degradation of LIN28B mRNA	Kf64*[mLin28B], kf64=0.008 min^{-1}	Estimated from (3, 5)
V80	Degradation of LIN28B protein	Kf65*[Lin28B], kf65=0.005 min^{-1}	Estimated from (3)
**No.**	**Reaction description**	**Reaction Rates and Parameters (k#, v#, n#)**	**Reference**
	**TGFβ activation of TSP-1 (Subpart B)**		
V81	Internalization of TGFβRI that activates SMAD1	Kf66*[TGFβR1$_{SMAD1}$]-kr66*[TGFβR1$_{SMAD1-INT}$], kf66=0.395 min^{-1}, kr66=0.0395 min^{-1}	(12)
V82	Internalization of TGFβRI that activates SMAD2	Kf66*[TGFβR1$_{SMAD2}$]-kr66*[TGFβR1$_{SMAD2-INT}$]	
V83	Internalization of TGFβRII	Kf66*[TGFβR2]-kr66*[TGFβR2$_{INT}$]	
V84	TGFβ signal activates calcium influx	Vm27*([Dimer$_{SMAD1-INT}$]^n27)/([Dimer$_{SMAD1-INT}$]^n27+kp27)*([Dimer$_{SMAD2-INT}$]^n28)/([Dimer$_{SMAD2-INT}$]^n28+kp28)*[in-switch], vm27=32 µM/min, n27=2, kp27=2.8e-9 $µM^2$, n28=2, kp28=3e-9 $µM^2$, see calcium regulation below for details of [in-switch]	Fitted
V85	Ca^{2+} binds calmodulin	Kf67*[Ca]*[CaM] – kr67*[CaM-Ca], kf67=1.1 $µM^{-1}min^{-1}$, kr67=12 min^{-1}	Estimated from (13)
V86	Activated calmodulin binds calcineurin	Kf68*[CaM-Ca]*[CaN] – kr68*[CaM-Ca-CaN], kf68=3.1 $µM^{-1}min^{-1}$, kr68=7.3 min^{-1}	Fitted
V87	Activated calcineurin dephosphorylates NFAT	Vm29*[pNFAT]*([CaM-Ca-CaN]^n29/([CaM-Ca-CaN]^n29+kp29)), vm29=0.01044 min^{-1}, n29=7, kp29=3e-9 $µM^7$	Fitted
V88	Phosphorylation of NFAT in cytoplasm	Kf69*[NFAT], kf69=0.06 min^{-1}	Estimated from (14)

V89	NFAT moves from nucleus to cytoplasm	Kf70*[$pNFAT_N$], kf70=0.2 min^{-1}	Estimated from (14)
V90	Phosphorylation of NFAT in nucleus	Kf69*[$NFAT_N$]	Estimated from (14)
V91	NFAT moves in to nucleus	Kf71*[NFAT], kf71=3 min^{-1}	Fitted
V92	Synthesis of TGFβRII	Vm30, vm30=1.33e-5 μM/min	(12)
V93	Degradation of TGFβRII	Kf72*[TGFβR2], kf72=0.0278 min^{-1}	(12)
V94	TGFβ binds TGFβRII	Kf73*[TGFβ]*[TGFβR2] –kr73*[TLR], kf73=397 $\mu M^{-1}min^{-1}$, kr73=0.298 min^{-1}	(12)
V95	LR complex dimerize with TGFβRI receptor that activates SMAD1	Kf73*[TLR]*[$TGF\beta R1_{SMAD1}$] – kr73*[$Dimer_{SMAD1}$]	
V96	LR complex dimerize with TGFβRI receptor that activates SMAD2	Kf73*[TLR]*[$TGF\beta R1_{SMAD2}$] – kr73*[$Dimer_{SMAD2}$]	
V97	Internalization of dimer activating SMAD1	Kf66*[$Dimer_{SMAD1}$]	
V98	Internalization of dimer activating SMAD2	Kf66*[$Dimer_{SMAD2}$]	
V99	Dimer activating SMAD1 binds SMAD1	Kf74* [$Dimer_{SMAD1-INT}$]*[SMAD1] – kr74*[SMAD1-$Dimer_{SMAD1-INT}$], kf74=19000 $\mu M^{-1}min^{-1}$, kr74=0.971 min^{-1}	Fitted; (12)
V100	Dimer activating SMAD2 binds SMAD2	Kf74* [$Dimer_{SMAD2-INT}$]*[SMAD2] – kr74*[SMAD2-$Dimer_{SMAD2-INT}$]	
V101	Phosphorylation of SMAD1	Kf75*[SMAD1-$Dimer_{SMAD1-INT}$], kf75=44800 min^{-1}	(12)
V102	Phosphorylation of SMAD2	Kf75*[SMAD2-$Dimer_{SMAD2-INT}$]	
V103	Shuttling of phosphorylated SMAD1 into nucleus	Kf76*[pSMAD1], kf76=0.503 min^{-1}	(12)

V104	Shuttling of phosphorylated SMAD2 into nucleus	Kf76*[pSMAD2]	
V105	Phosphorylated SMAD1 binds SMAD4	Kf77*[pSMAD1]*[SMAD4] – kr77*[pSMAD1-SMAD4], kf77=3600 $\mu M^{-1} min^{-1}$, kr77=1460 min^{-1}	Fitted; (12)
V106	Phosphorylated SMAD2 binds SMAD4	Kf77*[pSMAD2]*[SMAD4] – kr77*[pSMAD2-SMAD4]	
V107	pSMAD1-SMAD4 shuttling into nucleus	Kf78*[pSMAD1-SMAD4], kf78=0.8 min^{-1}	(12)
V108	pSMAD2-SMAD4 shuttling into nucleus	Kf78*[pSMAD2-SMAD4]	
V109	SMAD4 shuttling into nucleus	Kf79*[SMAD4] – kr79*[$SMAD4_N$], kf79=0.0201 min^{-1}, kr79=0.174 min^{-1}	(12)
V110	pSMAD1 binds SMAD4 in nucleus	Kf80*[$SMAD4_N$]*[$pSMAD1_N$]-kr80*[pSMAD1-$SMAD4_N$], kf80=100 $\mu M^{-1} min^{-1}$, kr80=0.909 min^{-1}	(12)
V111	pSMAD2 binds SMAD4 in nucleus	Kf80*[$SMAD4_N$]*[$pSMAD2_N$]-kr80*[pSMAD2-$SMAD4_N$]	
V112	Dephosphorylation of pSMAD1-SMAD4 in nucleus	Kf81*[pSMAD1-$SMAD4_N$],kf81=0.05802 min^{-1}	Fitted
V113	Dephosphorylation of pSMAD2-SMAD4 in nucleus	Kf82*[pSMAD2-$SMAD4_N$], kf82=0.01104 min^{-1}	Fitted
V114	Dephosphorylation of pSMAD1 in nucleus	Kf81*[$pSMAD1_N$]	
V115	Dephosphorylation of pSMAD2 in nucleus	Kf82*[$pSMAD2_N$]	
V116	Dissociation of SMAD1-SMAD4 in nucleus	Kf83*[SMAD1-$SMAD4_N$], kf83=0.101 min^{-1}	(12)
V117	Dissociation of SMAD2-SMAD4 in nucleus	Kf83*[SMAD2-$SMAD4_N$]	

V118	Shuttling of SMAD1 into nucleus	Kf84*[SMAD1]-kr84*[$SMAD1_N$], kf84=0.162 min^{-1}, kr84=0.348 min^{-1}	(12)
V119	Shuttling of SMAD2 into nucleus	Kf84*[SMAD2]-kr84*[$SMAD2_N$]	
V120	Synthesis of SMAD1	Vm31, vm31=4.55e-5 µM/min	(12)
V121	Synthesis of SMAD2	Vm31	
V122	Degradation of SMAD1	Kf85*[SMAD1], kf85=8.46e-4 min^{-1}	Estimated from (15)
V123	Degradation of SMAD2	Kf85*[SMAD2]	
V124	Dissociation of receptor dimer that activates SMAD1	Kr66*[$Dimer_{SMAD1-INT}$]	
V125	Dissociation of receptor dimer that activates SMAD2	Kr66*[$Dimer_{SMAD2-INT}$]	
V126	Synthesis of receptor that activates SMAD1	Vm30	
V127	Synthesis of receptor that activates SMAD2	Vm30	
V128	Degradation of receptor that activates SMAD1	Kf72*[$TGF\beta R_{SMAD1}$]	
V129	Degradation of receptor that activates SMAD2	Kf72*[$TGF\beta R_{SMAD2}$]	
V130	R-SMADs activates SMAD7	Vm32*(vm33+[pSMAD1-$SMAD4_N$]^n32/(kp32+[pSMAD1-$SMAD4_N$]^n32)+[pSMAD2-$SMAD4_N$]^n33/(kp33+[pSMAD2-$SMAD4_N$]^n33)), vm32=0.005 µM/min, vm33=1e-4, n32=2, kp32=0.1 μM^2, n33=2, kp33=0.02 μM^2	Estimated from (6)

V131	Degradation of SMAD7 mRNA	Kf86*[mSMAD7], kf86=0.001 min^{-1}	Estimated from (3, 5)
V132	SMAD7 protein translation	Kf87*[mSMAD7], kf87=0.5 min^{-1}	Estimated from (3)
V133	Degradation of SMAD7 protein	Kf88*[SMAD7], kf88=0.00588 min^{-1}	Estimated from (3)
V134	SMAD7 promotes SMAD4 degradation	[SMAD4]*(vm34+vm35*[SMAD7]/([SMAD7]+kp35)), vm34=0.0012 min^{-1}, vm35=0.0035 min^{-1}, kp35=1 µM	Vm34 estimated from (3); fitted
V135	SMAD4 synthesis	Vm36, vm36=8.3056e-5 µM/min	(12)
V136	SMAD7 sequesters SMAD1-receptor complex	Kf89*[SMAD7]*[SMAD1-$Dimer_{SMAD1\text{-}INT}$]-kr89*[SMAD7-SMAD1-$Dimer_{SMAD1INT}$], kf89=300 $\mu M^{-1}min^{-1}$, kr89=0.024 min^{-1}	Estimated from (4)
V137	SMAD7 sequesters SMAD2-receptor complex	Kf89*[SMAD7]*[SMAD2-$Dimer_{SMAD2\text{-}INT}$]-kr89*[SMAD7-SMAD2-$Dimer_{SMAD2\text{-}INT}$]	
V138	Calcium outflux	[Ca]*kf90*[out-switch], kf90=0.03 min^{-1}, see calcium regulation below for details of [out-switch]	Fitted

Calcium regulation (rule-based):

$[out\text{-}switch]_0$=0 (unitless), $[in\text{-}switch]_0$=1 (unitless), $[track]_0$=0 (unitless);

[out-switch]=0, [in-switch]=1, [track]=0 if [Ca]<=0.025 µM

[out-switch]=([Ca]-0.025)*54, [in-switch]=2e-5, [track]=1 if ([Ca]>0.12 and [track]<1)

[out-switch]=([Ca]-0.025)*54, [in-switch]=2e-5, [track]=1 if ([Ca]>0.025 and [track]>0)

[out-switch]=([Ca]-0.025)*2000, [track]=0 if ([Ca]>0.025 and [track]<1)

Results of sample calcium kinetics are shown in S1_Fig.

S1_Table. Reaction descriptions, rules, reaction rates and kinetic parameters of TSP-1 model. Reactions are formulated based on experimental evidence in the literature. Reaction rates v# here match with the numbers in the model diagram provided in the article. $Species_N$ represents that the species is in the nucleus, other species, assumed in protein or miR form, are in the cytoplasm; mSpecies represents the mRNA of the species. The values of some rate constants are taken or estimated (and then optimized) from previous studies and experimental measurements as denoted by the Ref#. The values of all the other rate constants shown in this table are obtained after optimization against published EC data.

S2_Table: Differential equations and species initial conditions

Species	Differential Equations ($\frac{d[Species]}{dt}$)	Species Initial Condition (μM)
	**Intracellular TSP-1 regulation (Subpart A)**	
HIF1,2α	v1-v2-v3-v7	0.2077, 0.2958
HIF1,2α-FIH complex	v3-v11	6.808e-4, 0.2532
HIF1,2α-PHD complex	v7-v12	0.0158, 0.0225
FIH-O_2-Fe-DG	v4+v11-v3	0.8824
O_2	-v4-v8	Nx(21% O_2):209, Hx (2% O_2):19.9
FIH-DG-Fe	v5-v4	0.2712
DG	-v5-v9	999
FIH-Fe	v6-v5	8.73e-3
Fe	-v6-v10	49.6
FIH	-v6	4.396e-4
HIF1,2α/OH	v11+v12-v13	0.00148, 0.0047
VHL	v14-v13	1.116
HIF1,2α/OH-VHL	v13-v14	0.03011, 0.00471
PHD2-O_2-Fe-DG	v12+v8-v7	0.789
PHD2-Fe-DG	v9-v8	0.949
PHD2-Fe	v10-v9	0.0569
PHD2	-v10	2.29e-3
$HIF1\alpha_N$	v2-v18	0.0577
$HIF2\alpha_N$	v2-v19	0.0821
$HIF1\beta_N$	-v18-v19	0.8756
TTP	v17-v16	0.0700
mTTP	V22-v21	0.00127
$HIF1\text{-}dimer_N$	v18	0.0101
$HIF2\text{-}dimer_N$	v19	0.0144
MYC	v20-v30-v15	0.0161
MYC_N	v30	0.0322

mMXI1	v24-v25	9.36e-5
MXI1	v26-v27-v28	0.0234
$MXI1_N$	v28	0.0585
mLin28B	V31-v79	1.88e-5
Lin28B	V33-v80-v34	0.00113
$Lin28B_N$	V34-v35	0.0113
$Lin28B_N$-pri-$let7_N$	V35	0.0591
mPSAP	V32-v36	3.36e-5
PSAP	V37-v38-v39	0.03357
$PSAP_N$	V39	0.0839
mP53	V40-v41	6.58e-5
P53	V42-v43-v44	0.1468
$P53_N$	V44	0.0587
Pri-miR-18a	V29-v45	9.54e-4
Pre-miR-18a	V45-v46-v47	7.91e-6
miR-18a	V47-v48-v49	0.00221
miR-18a RISC	V49-v50+v51	0.00288
miR-18a RISC-mTSP1	V50-v51	0
mTSP1/p-body	V51-v52-v53	3.92e-5
Pri-let-7_N	v23-v54	6.55e-5
Pre-let-7	V54-v55-v56	5.76e-6
Let-7	V56-v61-v62	2.506e-4
AGO1	V60-v57-v62-v49	0.7767
Let-7 RISC	V62-v63-v69+v64+v70	0.00278
mAGO1/p-body	V64-v65-v66	0.01795
mAGO1	V59-v58-v63+v66	0.00127
Let-7 RISC-mAGO1	V63-v64	6.29e-5
mDicer	V67-v68-v69+v72	2.93e-3
Let-7 RISC-mDicer	V69-v70	2.98e-5
mDicer/p-body	V70-v71-v72	0.0265
Dicer	V73-v74	1.0476
mTSP1	V75-v77-v50+v53	2.36e-5
TSP1	V76-v78	0.00867
	**TGFβ activation of TSP-1 (Subpart B)**	
$TGF\beta R1_{SMAD1}$	V124+v126-v81-v95-v128	4.78e-4
$TGF\beta R1_{SMAD1-INT}$	V81	0.0048
$TGF\beta R1_{SMAD2}$	V127+v125-v96-v129-v82	4.78e-4
$TGF\beta R1_{SMAD2-INT}$	V82	0.0048
TGFβR2	V92+v124+v125-v93-v94-v83	4.78e-4
$TGF\beta R2_{INT}$	V83	0.0048
TGFβ	-v94	0
TLR	V94-v95-v96	0
$Dimer_{SMAD1}$	V95-v97	0
$Dimer_{SMAD2}$	V96-v98	0
$Dimer_{SMAD1-INT}$	V97-v99+v101	0
$Dimer_{SMAD2-INT}$	V98-v100+v102	0

SMAD1- $Dimer_{SMAD1-INT}$	V99-v101	0
SMAD2- $Dimer_{SMAD2-INT}$	V100-v102	0
SMAD1	V120-v122-v99-v128	0.0538
SMAD2	V121-v123-v100-v129	0.0538
pSMAD1	V101-v103-v105	0
pSMAD2	V102-v104-v106	0
SMAD4	V135-v134-v105-v106-v109	0.0619
pSMAD1-SMAD4	V105-v107	0
pSMAD2-SMAD4	V106-v108	0
$pSMAD1_N$	V103-v114-v110	0
$pSMAD2_N$	V104-v115-v111	0
$SMAD4_N$	V109-v110-v111+v116+v117	0.0072
pSMAD1-$SMAD4_N$	V110-v112	0
pSMAD2-$SMAD4_N$	V111-v113	0
SMAD1-$SMAD4_N$	V112-v116	0
SMAD2-$SMAD4_N$	V113-v117	0
$SMAD1_N$	V114+v116+v118	0.025
$SMAD2_N$	V115+v117+v119	0.025
mSMAD7	V130-v131	5e-4
SMAD7	V132-v133-v136-v137	0.0425
SMAD7-SMAD1-$Dimer_{SMAD1-INT}$	V136	0
SMAD7-SMAD2-$Dimer_{SMAD2-INT}$	V137	0
Ca	V84-v85-v138, see calcium regulation in S1_Table	0.0254
CaM	-V85	5.9233
CaM-Ca	V85-v86	0.0138
CaN	-v86	4.9388
CaM-Ca-CaN	V86	0.0289
pNFAT	V89+v88-v87	1.5022
NFAT	V87-v88-v91	2.87e-5
$NFAT_N$	V91-v90	0.001435
$pNFAT_N$	V90-v89	4.31e-4

S2_Table. Model differential equations and species initial conditions of TSP-1 model. Initial conditions here refer to the steady-state (normoxia) concentration of each species in ECs without TGFβ treatment. To maintain a moderate complexity, the model assumes that transcription factors or enzymes in Hill-type (Michaelis-Menten) reactions are unconsumed, and mRNAs are unconsumed in translation. Values of initial conditions used in the model (e.g. oxygen sensing, TGFβ signaling) are either estimated (following by optimization against published EC data) or taken from literature (2, 12).

S1_Fig

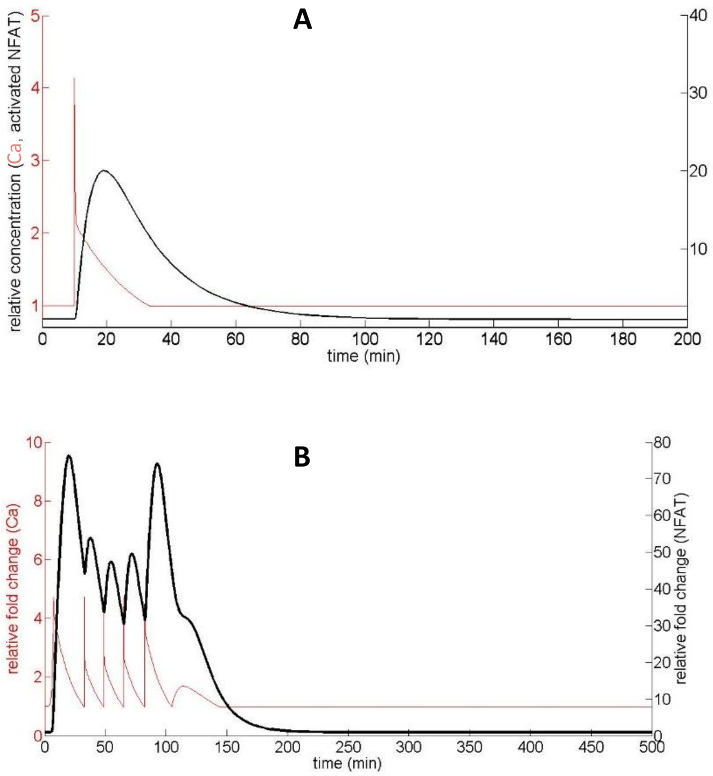

S1_Fig. Model of TGFβ-induced calcium regulation and downstream activation of NFAT in ECs. Multiple rule-based reactions are responsible for the TGFβ-induced calciumn regulation in the model. An assumption is that rate of calcium influx is dependent on the concentration of internalized TGFβ receptors that have bound ligands. The rules define that when the intracellular free calcium concentration reaches a threshold (~120 nM), calcium influx is significantly reduced and outflux rate is increased. The outflux rate is proportional to the difference between current intracelluar free calcium concentration and the baseline concentartion. When the intracelluar free calcium concentration is below the baseline level, rate of calcium influx and outflux is restored. The mathematical equations defining the rules are shown in S1_Table. (A-B) The resulting calcium profiles (red curve) in response to TGFβ treatment display a periodic behavior. Simulated NFAT (activated) dynamics (black curves, normalized in terms of relative fold changes) exhibit a stair-case like behavior which resembles the experimental data measured in (16).

S2_Fig

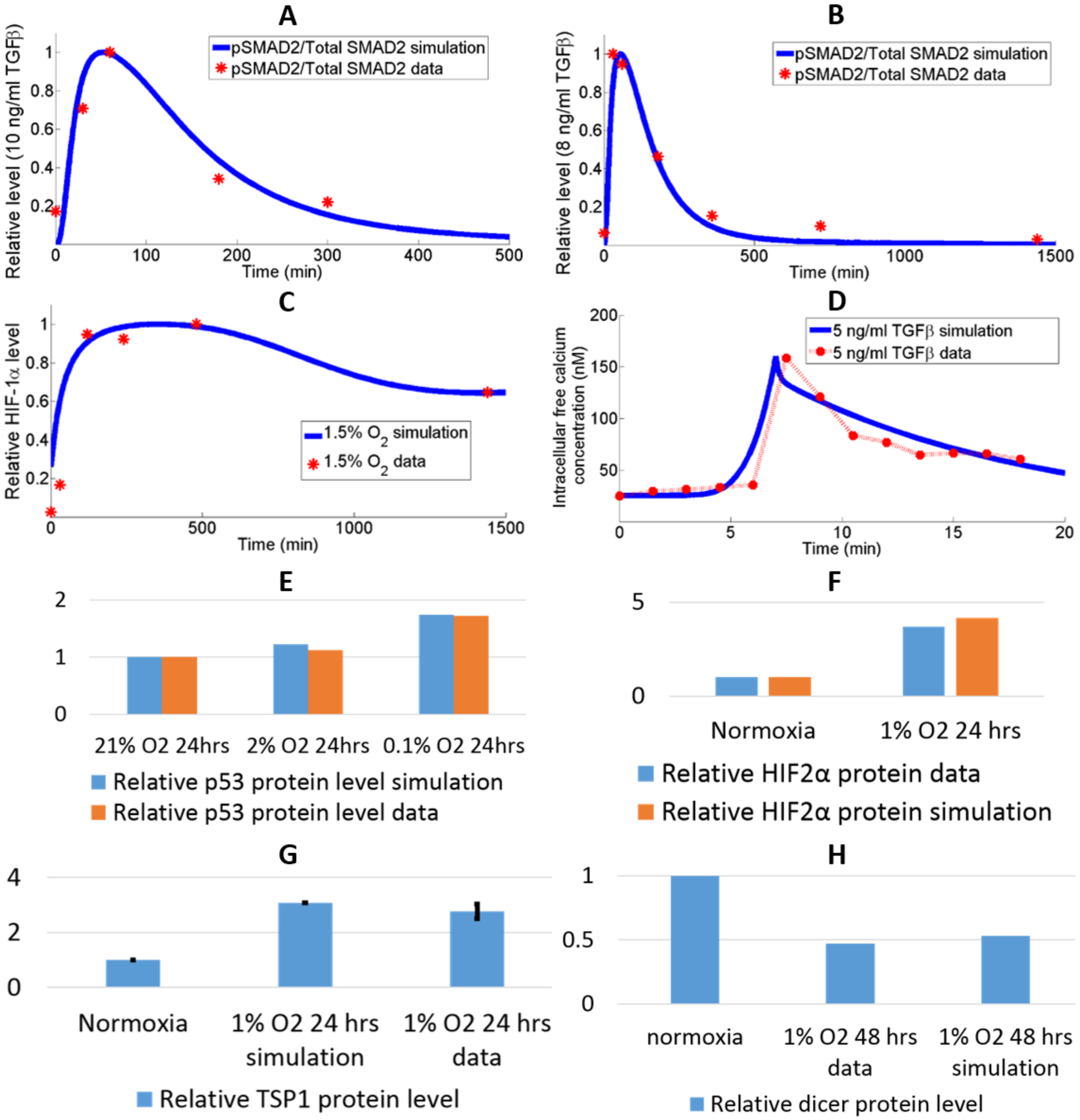
A
Relative level (10 ng/ml TGFβ)
pSMAD2/Total SMAD2 simulation
pSMAD2/Total SMAD2 data
Time (min)
B
Relative level (8 ng/ml TGFβ)
pSMAD2/Total SMAD2 simulation
pSMAD2/Total SMAD2 data
Time (min)
C
Relative HIF-1α level
1.5% O2 simulation
1.5% O2 data
Time (min)
D
Intracellular free calcium concentration (nM)
5 ng/ml TGFβ simulation
5 ng/ml TGFβ data
Time (min)
E
21% O2 24hrs
2% O2 24hrs
0.1% O2 24hrs
Relative p53 protein level simulation
Relative p53 protein level data
F
Normoxia
1% O2 24 hrs
Relative HIF2α protein data
Relative HIF2α protein simulation
G
Normoxia
1% O2 24 hrs simulation
1% O2 24 hrs data
Relative TSP1 protein level
H
normoxia
1% O2 48 hrs data
1% O2 48 hrs simulation
Relative dicer protein level

S2_Fig. Additional model calibration against published fibroblast data using different paramter values. Simulation and data of (A) relative ratio of phosphorylated SMAD2 protein in response to 10 ng/ml TGFβ1 in human lung myofibroblasts (17), (B) relative ratio of phosphorylated SMAD2 protein in response to 8 ng/ml TGFβ1 in human normal primary fibroblasts (18), (C) relative level of HIF1α protein in 1.5% O_2 in human embryonic fibroblasts (19), (D) concentrations of intracelluar free calcium in response to 5 ng/ml TGFβ1 treatment over twenty minutes (20), (E) relative p53 protein levels in different oxygen tensions in normal human lung fibroblasts (21), (F-G) relative HIF2α and TSP-1 protein levels in hypoxia (1% O_2) in pulmonary-derived murine fibroblasts (22), (H) relative Dicer protein level in response to hypoxia (1% O_2) in mouse embryonic fibroblasts (23). (A-H) The primary goal of these additional validations is to show that our model, which is originally based in ECs, has the potential to be applied to simulate TSP-1 pathway signals in fibroblasts. Aiming for the minimum amount of changes in the EC paramter set, values of three parameters are altered here in order to fit the data in fibroblasts: kf74 is set to 1200 $\mu M^{-1} min^{-1}$, maximum intracelluar calcium allowed is set to 0.16 μM, and kp1 is set to 0.023 μM^4.

S3_Fig

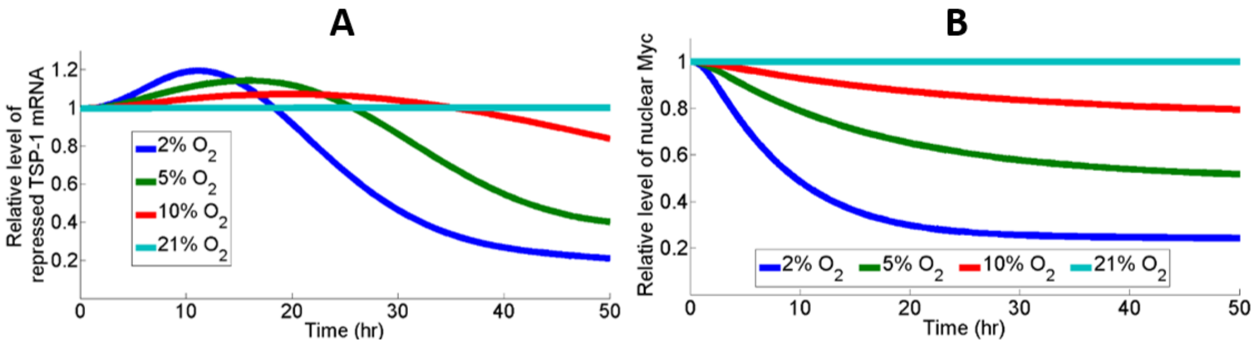

S3_Fig. De-suppression of TSP-1 mRNA and downregulation of Myc in hypoxia. In response to hypoxic stresses, the model predicts (A) a significant reduction in the amount of TSP-1 mRNAs that are suppressed by miRs. The initial rises in the simulation curves are due the rapid increase in hypoxia-driven TSP-1 transcriptional activation and the delayed, gradual downregulation of miR-18a. (B) Hypoxia promotes Myc protein degradation and results in its downregulation. Decrease in the abundance of Myc leads to a drop in the expression of miR-18a because of reduced transcriptional activation.

S4_Fig

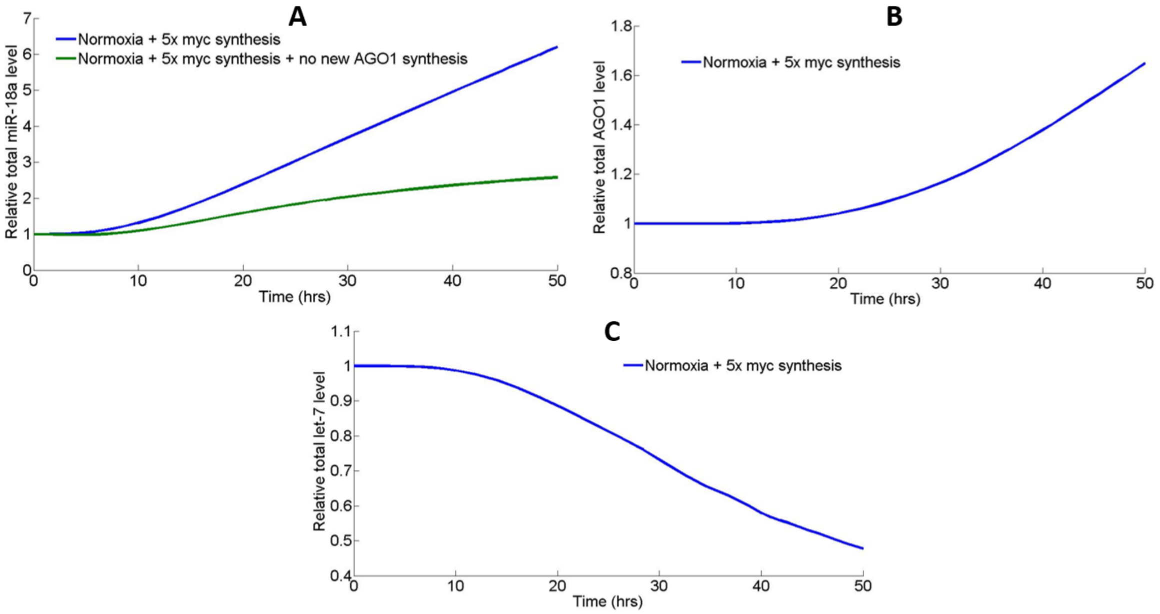

S4_Fig. AGO1 upregulation following myc overexpression. (A) Hyperactive Myc activity results in an increased level of miR-18a, which is partially contributed by (B) an increase in AGO1 level. (C) AGO1 upregulation is a result of let-7 downregulation by the Myc-Lin28B-let7 axis.

S5_Fig

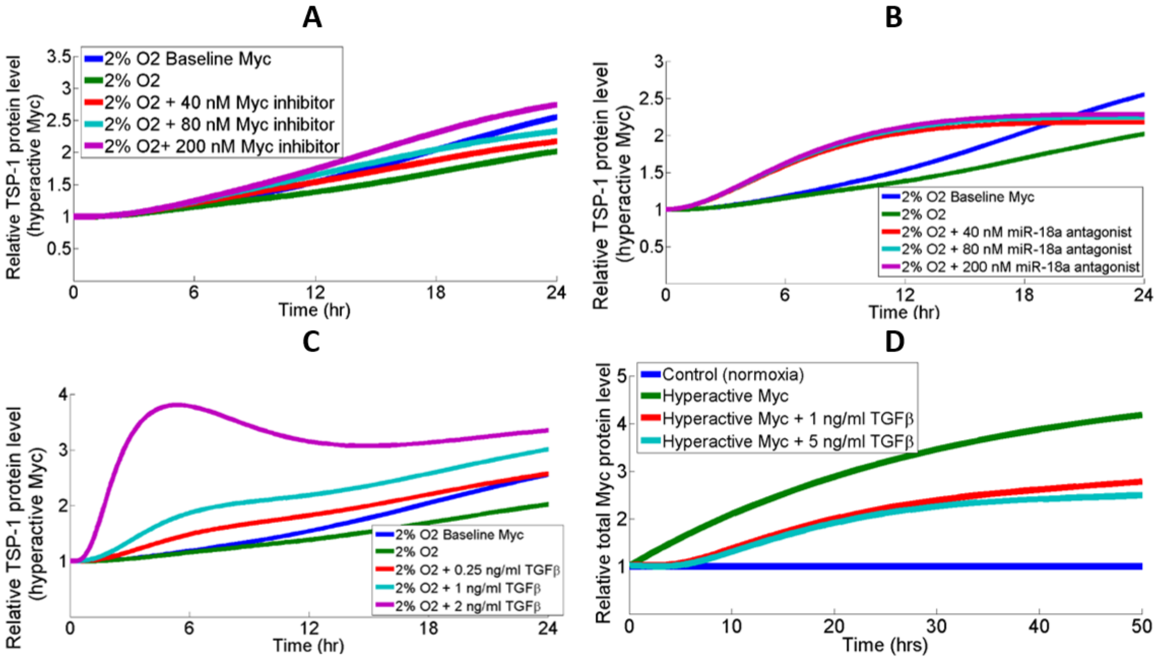

S5_Fig. Testing different therapeutic strategies in hypoxia and hyperactive Myc conditions. Simulations of TSP-1 protein expression in response to different doses of (A) Myc inhibitor, (B) miR-18a antagonist, and (C) TGFβ under the condition of Myc hyperactivity (Myc synthesis rate multiplied by 5) and hypoxia (2% Oxygen). Unlike the results presented in Fig 7, here TGFβ stimulation is predicted to be more effective compared to the other two strategies throughout a 24-hour simulation timespan, which agrees with the sensitivity analysis results shown in Fig 9. (D) TGFβ treatment not only induces TSP-1 transcription directly but also downregulates Myc expression in scenarios of hyperactive Myc (control is baseline Myc, and simulations are in normoxia). (A-D) Results are normalized with respect to the normoxic steady state value computed with baseline Myc activity.

S6_Fig

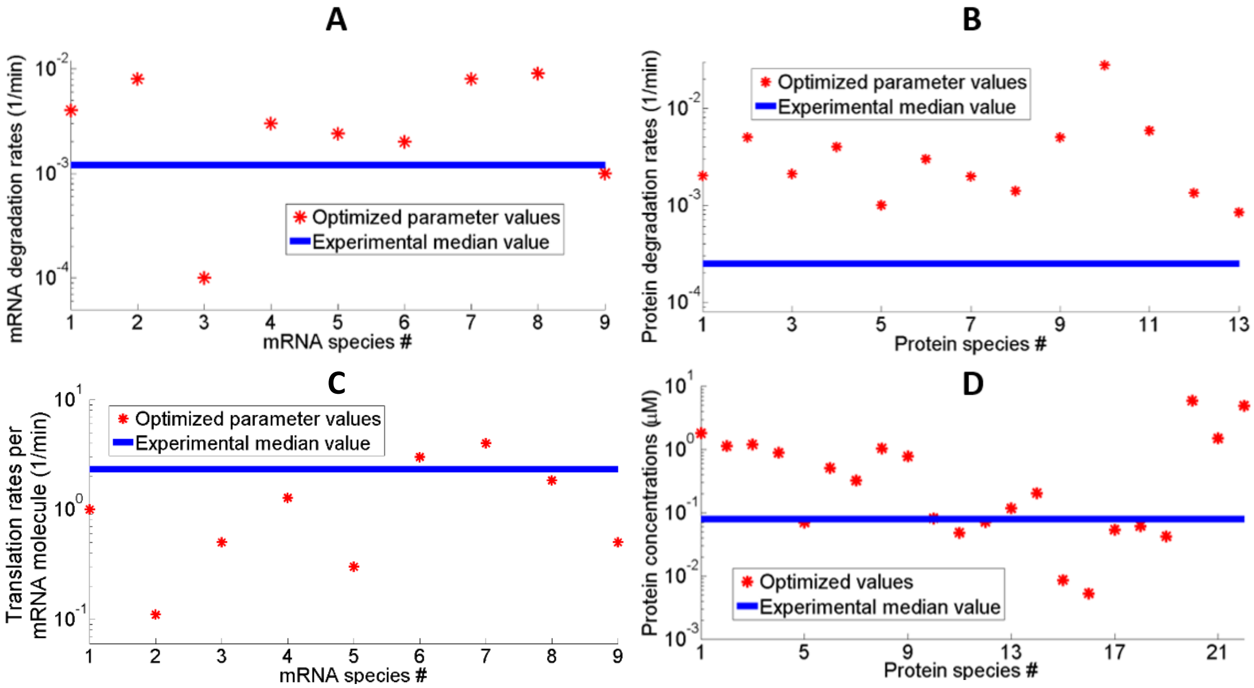

S6_Fig. Model parameter distributions. Distribution of protein/mRNA turnover parameters and initial conditions used in the model including (A) mRNA degradation rates of different species (median 1.2e-3 min^{-1}), (B) protein degradation rates of different species (median 2.5e-4 min^{-1}), (C) rate of translation of different mRNA species (median 2.33 proteins per mRNA per min), and (D) protein concentrations of different species (median 0.08 µM). (A-D) The red asterisks are optimized model parameter values and the blue lines are the experimental median values obtained from literature (3, 5).

S7_Fig

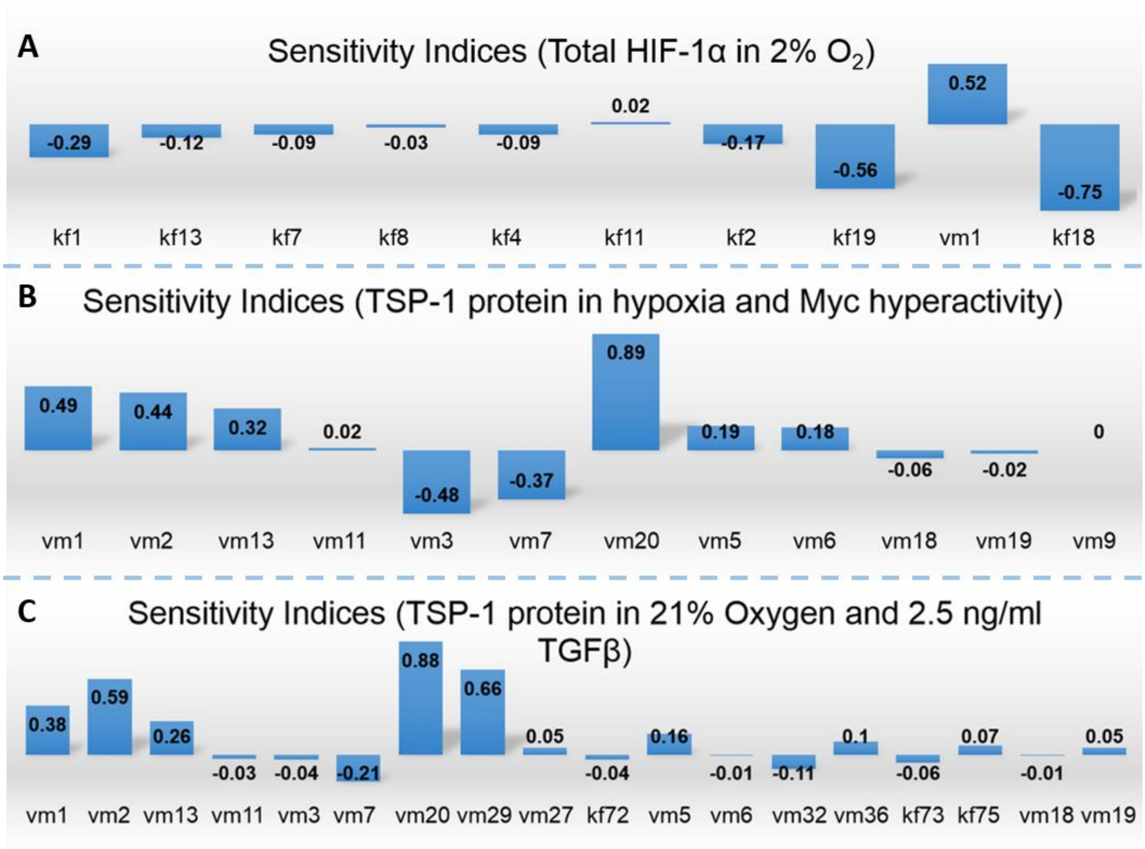

S7_Fig. Additional sensitivity analysis. AUCs of (A) total HIF-1α in 48 hours and (B-C) TSP-1 protein in 24 hours under different simulated conditions. (A-C) Rate descriptions—kf1: HIF-1α translocation into nucleus; kf2: HIF-1α binds FIH complex; kf4: oxygen binds FIH complex; kf7: HIF-1α binds PHD complex; kf8: oxygen binds PHD complex; kf11: HIF1α-OH-FIH dissociation; kf13: HIF1α-OH-PHD dissociation; kf18: TTP synthesis; kf19: HIF-1α binds HIF-1β; vm1: HIF-1α synthesis; vm2: HIF-2α synthesis; vm3: Myc synthesis; vm5: let-7 synthesis; vm6: MXI-1 synthesis; vm7: miR-18a synthesis; vm9: LIN28B synthesis; vm11: PSAP synthesis; vm13: p53 synthesis; vm18: Ago1 synthesis; vm19: Dicer synthesis; vm20: TSP-1 synthesis; kf72: TGFβR degradation; kf73: TGFβ binds its receptor; kf75: R-SMAD phosphorylation; vm27: TGFβ-mediated calcium influx; vm29: NFAT dephosphorylation; vm32: SMAD7 synthesis; vm36: SMAD4 synthesis.

References

1. Li GW, Burkhardt D, Gross C, Weissman JS. Quantifying absolute protein synthesis rates reveals principles underlying allocation of cellular resources. Cell. 2014;157(3):624-35.
2. Qutub AA, Popel AS. A computational model of intracellular oxygen sensing by hypoxia-inducible factor HIF1 alpha. Journal of cell science. 2006;119(Pt 16):3467-80.
3. Schwanhausser B, Busse D, Li N, Dittmar G, Schuchhardt J, Wolf J, et al. Corrigendum: Global quantification of mammalian gene expression control. Nature. 2013;495(7439):126-7.
4. Yugandhar K, Gromiha MM. Protein-protein binding affinity prediction from amino acid sequence. Bioinformatics. 2014;30(24):3583-9.
5. Yang E, van Nimwegen E, Zavolan M, Rajewsky N, Schroeder M, Magnasco M, et al. Decay rates of human mRNAs: correlation with functional characteristics and sequence attributes. Genome research. 2003;13(8):1863-72.
6. Miller C, Schwalb B, Maier K, Schulz D, Dumcke S, Zacher B, et al. Dynamic transcriptome analysis measures rates of mRNA synthesis and decay in yeast. Molecular systems biology. 2011;7:458.
7. Yang X, Li H, Huang Y, Liu S. The dataset for protein-RNA binding affinity. Protein science : a publication of the Protein Society. 2013;22(12):1808-11.
8. Gantier MP, McCoy CE, Rusinova I, Saulep D, Wang D, Xu D, et al. Analysis of microRNA turnover in mammalian cells following Dicer1 ablation. Nucleic acids research. 2011;39(13):5692-703.
9. Klironomos FD, Berg J. Quantitative analysis of competition in posttranscriptional regulation reveals a novel signature in target expression variation. Biophysical journal. 2013;104(4):951-8.
10. Gokhale SA, Gadgil CJ. Analysis of miRNA regulation suggests an explanation for 'unexpected' increase in target protein levels. Molecular bioSystems. 2012;8(3):760-5.
11. Riley KJ, Yario TA, Steitz JA. Association of Argonaute proteins and microRNAs can occur after cell lysis. Rna. 2012;18(9):1581-5.
12. Nicklas D, Saiz L. Computational modelling of Smad-mediated negative feedback and crosstalk in the TGF-beta superfamily network. Journal of the Royal Society, Interface / the Royal Society. 2013;10(86):20130363.
13. Crouch TH, Klee CB. Positive cooperative binding of calcium to bovine brain calmodulin. Biochemistry. 1980;19(16):3692-8.
14. Shen T, Cseresnyes Z, Liu Y, Randall WR, Schneider MF. Regulation of the nuclear export of the transcription factor NFATc1 by protein kinases after slow fibre type electrical stimulation of adult mouse skeletal muscle fibres. The Journal of physiology. 2007;579(Pt 2):535-51.
15. Lo RS, Massague J. Ubiquitin-dependent degradation of TGF-beta-activated smad2. Nature cell biology. 1999;1(8):472-8.
16. Noren DP, Chou WH, Lee SH, Qutub AA, Warmflash A, Wagner DS, et al. Endothelial cells decode VEGF-mediated Ca2+ signaling patterns to produce distinct functional responses. Science signaling. 2016;9(416):ra20.
17. Roach KM, Feghali-Bostwick C, Wulff H, Amrani Y, Bradding P. Human lung myofibroblast TGFbeta1-dependent Smad2/3 signalling is Ca(2+)-dependent and regulated by KCa3.1 K(+) channels. Fibrogenesis & tissue repair. 2015;8:5.
18. Li Q, Zhang D, Wang Y, Sun P, Hou X, Larner J, et al. MiR-21/Smad 7 signaling determines TGF-beta1-induced CAF formation. Scientific reports. 2013;3:2038.
19. Poulios E, Trougakos IP, Gonos ES. Comparative effects of hypoxia on normal and immortalized human diploid fibroblasts. Anticancer research. 2006;26(3A):2165-8.

20. Alevizopoulos A, Dusserre Y, Ruegg U, Mermod N. Regulation of the transforming growth factor beta-responsive transcription factor CTF-1 by calcineurin and calcium/calmodulin-dependent protein kinase IV. The Journal of biological chemistry. 1997;272(38):23597-605.
21. Mizuno S, Bogaard HJ, Voelkel NF, Umeda Y, Kadowaki M, Ameshima S, et al. Hypoxia regulates human lung fibroblast proliferation via p53-dependent and -independent pathways. Respiratory research. 2009;10:17.
22. Labrousse-Arias D, Castillo-Gonzalez R, Rogers NM, Torres-Capelli M, Barreira B, Aragones J, et al. HIF-2alpha-mediated induction of pulmonary thrombospondin-1 contributes to hypoxia-driven vascular remodelling and vasoconstriction. Cardiovascular research. 2016;109(1):115-30.
23. Rupaimoole R, Wu SY, Pradeep S, Ivan C, Pecot CV, Gharpure KM, et al. Hypoxia-mediated downregulation of miRNA biogenesis promotes tumour progression. Nature communications. 2014;5:5202.
