# Supplementary material for: Transcriptional and Post-Transcriptional Regulation of Thrombospondin-1 Expression: A Computational Model
Source: PLoS Comput Biol. 2017 Jan 3;13(1):e1005272. doi: 10.1371/journal.pcbi.1005272 (PMC5207393; doi:10.1371/journal.pcbi.1005272)
Supplement: S1 Table — (PDF) [file pcbi.1005272.s002.pdf]

S1_Table: Reaction descriptions, reaction rates, kinetic parameters of TSP-1 model

No.	Reaction description	Reaction Rates and Parameters (k#, v#, n#)	Reference
	**Intracellular TSP-1 regulation (Subpart A)**		
V1	Synthesis of HIFs	HIF1a: vm1*(1-[TTP]^n1/(kp1+[TTP]^n1)), vm1=0.03012 μM/min, n1=4, kp1=0.093 μM^4, HIF2a: vm2, vm2=0.096 μM/min	Vm1 and vm2 estimated from (1); fitted
V2	HIF shuttling into nucleus	HIF1a: kf1*[HIF1a] - kr1*[$HIF1a_N$], kf1=0.005 min^{-1}, kr1=0.018 min^{-1}, HIF2a: kf1*[HIF2a] – kr1*[$HIF2a_N$]	Fitted
V3	HIF binding with FIH complex	HIF1a: kf2*[HIF1a]*[FIH-O2-Fe-DG] – kr2*[HIF1a-FIH complex], kf2=0.13 $\mu M^{-1}min^{-1}$, kr2=1 min^{-1}, HIF2a: kf3*[HIF2a]*[FIH-O2-Fe-DG] – kr2*[HIF2a-FIH complex], kf3=1.3 $\mu M^{-1}min^{-1}$	Kf2 and kr2 estimated from (2); fitted
V4	Oxygen binding with FIH	kf4*[O2]*[FIH-DG-Fe] – kr4*[FIH-O2-Fe-DG], kf4=0.165 $\mu M^{-1}min^{-1}$, kr4=10.6 min^{-1}	Fitted; estimated from (2)
V5	2-OG binding with FIH	kf5*[FIH-Fe]*[DG] – kr5*[FIH-DG-Fe], kf5=0.23 $\mu M^{-1}min^{-1}$, kr5=7.4 min^{-1}	Estimated from (2)
V6	Iron binding with FIH	kf6*[Fe]*[FIH] – kr6*[FIH-Fe], kf6=4 $\mu M^{-1}min^{-1}$, kr6=10 min^{-1}	Fitted
V7	HIF binding with PHD complex	HIF1a: kf7*[PHD2-O2-Fe-DG]*[HIF1a] – kr7*[HIF1a-PHD complex], kf7=0.11 $\mu M^{-1}min^{-1}$, kr7=0.7 min^{-1}, HIF2a: kf7*[PHD2-O2-Fe-DG]*[HIF2a] – kr7*[HIF2a-PHD complex]	(2)
V8	Oxygen binding with PHD	kf8*[O2]*[PHD2-O2-Fe-DG] – kr8*[PHD2-O2-Fe-DG], kf8=0.043 $\mu M^{-1}min^{-1}$, kr8=10.8 min^{-1}	(2)
V9	2-OG binding with PHD	kf9*[DG]*[PHD2-Fe] – kr9*[PHD2-Fe-DG], kf9=0.18 $\mu M^{-1}min^{-1}$, kr9=10.8 min^{-1}	(2)
V10	Iron binding with PHD	kf10*[PHD2]*[Fe] – kr10*[PHD2-Fe], kf10=18 $\mu M^{-1}min^{-1}$, kr10=36 min^{-1}	(2)
V11	Hydroxylation of HIF-FIH complex	HIF1a: kf11*[HIF1a-FIH complex], kf11=34 min^{-1}, HIF2a: kf12*[HIF2a-FIH complex], kf12=0.34 min^{-1}	Fitted; estimated from (2)
V12	Hydroxylation of HIF-PHD complex	HIF1a: kf13*[HIF1a-PHD complex], kf13=0.44 min^{-1}, HIF2a: kf13*[HIF2a-PHD complex]	(2)
V13	VHL binding with	HIF1a: kf14*[VHL]*[HIF1a-OH] – kr14*[HIF1a-OH-VHL], kf14=42 $\mu M^{-1}min^{-1}$, kr14=1.3 min^{-1},	(2)

	hydroxylated HIF	HIF2a: kf14*[VHL]*[HIF2a-OH] – kr14*[HIF2a-OH-VHL]	
V14	Degradation of HIF	HIF1a: kf15*[HIF1a-OH-VHL], kf15=1 $\mu M^{-1}min^{-1}$, HIF2a: kf15*[HIF2a-OH-VHL]	Fitted
V15	HIF1a promotes myc degradation	[MYC]*(kf16+vm2*([HIF1a]^n2/([HIF1a]^n2+kp2))), kf16=0.001 min^{-1}, vm2=0.05 min^{-1}, n2=2, kp2=3 μM^2	Kf16 estimated from (3); fitted
V16	Degradation of TTP protein	kf17*[TTP], kf17=0.002 min^{-1}	Estimated from (3)
V17	Protein translation of TTP	kf18*[mTTP], kf18=0.11 min^{-1}	Estimated from (3)
V18	Association between HIF1-α and HIF1-β	kf19*[HIF1b]*[$HIF1a_N$] - kr19*[$HIF1\text{-}dimer_N$], kf19=0.006 $\mu M^{-1}min^{-1}$, kr19=0.03 min^{-1}	Estimated from (4)
V19	Association between HIF2-α and HIF1-β	kf19*[$HIF2a_N$]*[HIF1b] - kr19*[$HIF2\text{-}dimer_N$]	
V20	SMAD inhibits Myc production	vm3*(1-[$psmad2\text{-}smad4_N$]/([$psmad2\text{-}smad4_N$]+kp3)), vm3=2.75e-5 µM/min, kp3=0.004 µM	Estimated from (1); fitted
V21	Degradation of TTP mRNA	kf20*[mTTP], kf20=0.004 min^{-1}	Estimated from (3, 5)
V22	HIF1 activates TTP production	vm4*([$HIF1\text{-}dimer_N$]^n4) /(kp4+([$HIF1\text{-}dimer_N$]^n4)), vm4=5e-5 µM/min, n4=2, kp4=9e-4 μM^2	Estimated from (6)
V23	HIF1 activates let-7	Vm5*([$HIF1\text{-}dimer_N$]^n5/(kp5+[$HIF1\text{-}dimer_N$]^n5)), vm5=1.0607e-4 µM/min, n5=3, kp5=5.181e-5 μM^3	Estimated from (6)
V24	HIF1 activates MXI-1	Vm6*[$HIF1\text{-}dimer_N$]^n6/([$HIF1\text{-}dimer_N$]^n6+kp6), vm6=6e-6 µM/min, n6=2, kp6=6.25e-4 μM^2	Estimated from (6)
V25	Degradation of MXI-1 mRNA	Kf21*[mMXI1], kf21=0.009 min^{-1}	Estimated from (3, 5)
V26	Protein translation of MXI-1	Kf22*[mMXI1], kf22=1 min^{-1}	Estimated from (3)
V27	Degradation of MXI-1 protein	Kf23*[MXI1], kf23=0.004 min^{-1}	Estimated from (3)
V28	MXI-1 shuttling into the nucleus	Kf24*[MXI1] – kr24*[$MXI1_N$], kf24=0.01 min^{-1}, kr24=0.004 min^{-1}	Fitted
V29	Transcription of miR-18a	Vm7*(vm8+[MYC]^n7/([MYC]^n7+kp7)) *(1-[$MXI1_N$]^n8/([$MXI1_N$]^n8+kp8)),vm7=6.8708e-5 µM/min,vm8=0.02,n7=2, kp7=0.01211 μM^2, n8=2, kp8=0.2 μM^2	Estimated from (6)

V30	Myc shuttling into the nucleus	Kf25*[MYC] – kr25*[MYC_N], kf25=0.01 min^{-1}, kr25=0.005 min^{-1}	Fitted
V31	Transcription of Lin28B	Vm9*([MYC_N]^n9)/(kp9+[MYC_N]^n9) *([$MXI1_N$]/([$MXI1_N$]+kp10)), vm9=4.354e-6 μM/min, n9=2, kp9=0.0151 μM^2, kp10=0.05 μM	Estimated from (6)
V32	Transcription of PSAP	Vm11* (1-[MYC_N]^n11/([MYC_N]^n11+kp11)) *([$MXI1_N$]/([$MXI1_N$]+kp12)), vm11=3.688e-7 μM/min, n11=2, kp11=6.054e-4 μM^2, kp12=0.06 μM	Estimated from (6)
V33	Protein translation of Lin28B	Kf26*[mLin28B], kf26=0.3 min^{-1}	Estimated from (3)
V34	Lin28B shuttling into the nucleus	Kf27*[Lin28B] – kr27*[$Lin28B_N$], kf27=0.03 min^{-1}, kr27=0.003 min^{-1}	Fitted
V35	Lin28B sequesters pri-let-7	Kf28*[$Lin28B_N$]*[pri-let-7_N] – kr28*[$Lin28B_N$-pri-let-7_N], kf28=4000 $\mu M^{-1}min^{-1}$, kr28=min^{-1}	Estimated from (7)
V36	Degradation of PSAP mRNA	Kf29*[mPSAP], kf29=0.002 min^{-1}	Estimated from (3, 5)
V37	Protein translation of PSAP	Kf30*[mPSAP], kf30=3 min^{-1}	Estimated from (3)
V38	Degradation of PSAP protein	Kf31*[PSAP], kf31=0.003 min^{-1}	Estimated from (3)
V39	PSAP shuttling into the nucleus	Kf32*[PSAP] – kr32*[$PSAP_N$], kf32=0.05 min^{-1}, kr32=0.02 min^{-1}	Fitted
V40	PSAP activates P53	Vm13*([$PSAP_N$]/([$PSAP_N$]+kp13)), vm13=4.402e-7 μM/min, kp13=0.15 μM	Estimated from (6)
V41	Degradation of p53 mRNA	Kf33*[mP53], kf33=0.0024^{-1}	Estimated from (3, 5)
V42	Protein translation of p53	Kf34*[mP53], kf34=4 min^{-1}	Estimated from (3)
V43	HIF1a prevents p53 degradation	Vm14*[P53]*(1-[HIF1a] /([HIF1a] +kp14)), vm14=0.00198 min^{-1}, kp14=2 μM	Estimated from (3); fitted
V44	P53 shuttling in the nucleus	Kf35*[P53] – kr35*[$P53_N$], kf35=0.08 min^{-1}, kr35=0.2 min^{-1}	Fitted
V45	Cleavage of pri-miR-18a	Kf36*[pri-miR-$18a_N$], kf36=0.007 min^{-1}	Fitted
V46	Degradation of pre-miR-18a	Kf37*[pre-miR-18a], kf37=0.006 min^{-1}	Fitted
V47	Cleavage of pre-miR-18a	Vm15*[Dicer]*([pre-mir-18a]/([pre-mir-18a]+kp15)), vm15=0.04 min^{-1}, kp15=0.05 μM	Fitted

V48	Degradation of miR-18a	Kf38*[miR-18a], kf38=0.003 min^{-1}	Estimated from (8)
V49	miR-18a binding AGO1	Kf39*[AGO1]*[miR-18a] – kr39*[miR-18a RISC], kf39=1.84 $\mu M^{-1} min^{-1}$, kr39=1.098 min^{-1}	Estimated from (7, 9)
V50	miR-18a RISC binding TSP-1 mRNA	Kf40*[miR-18a RISC]*[mTSP1] – kr40*[miR-18a RISC-mTSP1], kf40=2 $\mu M^{-1} min^{-1}$, kr40=0.23 min^{-1}	Fitted
V51	TSP-1 mRNA to p-body	Kf41*[miR-18a RISC-mTSP1], kf41=0.6 min^{-1}	Estimated from (10)
V52	TSP-1 mRNA degradation in the p-body	Kf42*[mTSP1/p-body], kf42=5e-4 min^{-1}	Fitted
V53	TSP-1 mRNA return to cytoplasm	Kf43*[mTSP1/p-body], kf43=0.002 min^{-1}	Estimated from (10)
V54	Cleavage of pri-let-7	[pri-let-7_N]*vm16*[Let-7 RISC]^n16/([Let-7 RISC]^n16+kp16), vm16=5 min^{-1}, n16=2, kp16=0.001215 μM^2	Fitted
V55	Degradation of pre-let-7	Kf44*[pre-let-7], kf44=0.01 min^{-1}	Fitted
V56	Cleavage of pre-let-7	Vm17*[Dicer]*[pre-let-7] /(kp17+[pre-let-7]), vm17=0.01 min^{-1}, kp17=0.03 μM	Fitted
V57	Protein degradation AGO1	Kf45*[AGO1], kf45=0.0021 min^{-1}	Estimated from (3)
V58	AGO1 mRNA degradation	Kf46*[mAGO1], kf46=1e-4 min^{-1}	Estimated from (3, 5)
V59	AGO1 mRNA synthesis	Vm18, vm18=6e-7 μM/min	Estimated from (6)
V60	Protein translation of AGO1	Kf47*[mAGO1], kf47=1.275 min^{-1}	Estimated from (3)
V61	Degradation of let-7	Kf48*[let-7], kf48=0.00803 min^{-1}	Estimated from (8)
V62	Let-7 binds AGO1	Kf49*[let-7]*[AGO1] – kr49*[let-7 RISC], kf49=1 $\mu M^{-1} min^{-1}$, kr49=0.07 min^{-1}	Estimated from (7, 9, 11)
V63	Let-7 RISC binds AGO1 mRNA	Kf50*[let-7 RISC]*[mAGO1]-kr50*[let-7 RISC-mAGO1], kf50=8 $\mu M^{-1} min^{-1}$, kr50=0.15 min^{-1}	Fitted
V64	AGO1 mRNA to p-body	Kf51*[let-7 RISC-mAGO1], kf51=0.3 min^{-1}	Estimated from (10)
V65	AGO1 mRNA degradation in p-body	Kf52*[mAGO1/p-body], kf52=2.63e-5 min^{-1}	Fitted

V66	AGO1 mRNA return to cytoplasm	Kf53*[mAGO1/p-body], kf53=0.001025 min^{-1}	Estimated from (10)
V67	Dicer mRNA synthesis	Vm19, vm19=2.4e-5 µM/min	Fitted
V68	Dicer mRNA degradation	Kf54*[mDicer], kf4=0.008 min^{-1}	Estimated from (3, 5)
V69	Let-7 RISC binds Dicer mRNA	Kf55*[let-7 RISC]*[mDicer]-kr55*[let-7 RISC-mDicer], kf55=6.9 $\mu M^{-1} min^{-1}$, kr55=0.09 min^{-1}	Fitted
V70	Dicer mRNA to p-body	Kf56*[let-7 RISC-mDicer], kf56=1.8 min^{-1}	Estimated from (10)
V71	Dicer mRNA degradation in p-body	Kf57*[mDicer/p-body], kf57=2e-5 min^{-1}	Fitted
V72	Dicer mRNA return to cytoplasm	Kf58*[mDicer/p-body], kf58=0.001 min^{-1}	Estimated from (10)
V73	Dicer protein translation	Kf59*[mDicer], kf59=0.5 min^{-1}	Estimated from (3)
V74	Dicer protein degradation	Kf60*[Dicer], kf60=0.0014 min^{-1}	Estimated from (3)
V75	TSP1 synthesis	Vm20*(vm21+[psmad2-smad4$_N$] /([psmad2-smad4$_N$]+kp21)) *([P53$_N$] /([P53$_N$] +kp22)) *(vm23+[NFAT$_N$] /([NFAT$_N$]+kp23)) *(([HIF2-dimer$_N$])/([HIF2-dimer$_N$]+kp24))* (1-([MYC$_N$])/([MYC$_N$]+kp25)) *([MXI1$_N$]/([MXI1$_N$] +kp26)), vm20=5.18e-5 µM/min, vm21=0.1, kp21=0.0899 µM, kp22=0.1 µM, vm23=0.001, kp23=0.00926 µM, kp24=0.015, kp25=0.11 µM, kp26=0.005 µM	Estimated from (6)
V76	TSP1 protein translation	Kf61*[mTSP1], kf61=1.84 min^{-1}	Estimated from (3)
V77	TSP1 mRNA degradation	Kf62*[mTSP1], kf62=0.003 min^{-1}	Estimated from (3, 5)
V78	TSP1 protein degradation	Kf63*[TSP1], kf63=0.005 min^{-1}	Estimated from (3)
V79	Degradation of LIN28B mRNA	Kf64*[mLin28B], kf64=0.008 min^{-1}	Estimated from (3, 5)
V80	Degradation of LIN28B protein	Kf65*[Lin28B], kf65=0.005 min^{-1}	Estimated from (3)

No.	Reaction description	Reaction Rates and Parameters (k#, v#, n#)	Reference
	**TGFβ activation of TSP-1 (Subpart B)**		
V81	Internalization of TGFβRI that activates SMAD1	Kf66*[TGFβR1$_{SMAD1}$]-kr66*[TGFβR1$_{SMAD1-INT}$], kf66=0.395 min^{-1}, kr66=0.0395 min^{-1}	(12)
V82	Internalization of TGFβRI that activates SMAD2	Kf66*[TGFβR1$_{SMAD2}$]-kr66*[TGFβR1$_{SMAD2-INT}$]	
V83	Internalization of TGFβRII	Kf66*[TGFβR2]-kr66*[TGFβR2$_{INT}$]	
V84	TGFβ signal activates calcium influx	Vm27*([Dimer$_{SMAD1-INT}$]^n27)/([Dimer$_{SMAD1-INT}$]^n27+kp27)*([Dimer$_{SMAD2-INT}$]^n28)/([Dimer$_{SMAD2-INT}$]^n28+kp28)*[in-switch], vm27=32 µM/min, n27=2, kp27=2.8e-9 µM^2, n28=2, kp28=3e-9 µM^2, see calcium regulation below for details of [in-switch]	Fitted
V85	Ca^{2+} binds calmodulin	Kf67*[Ca]*[CaM] – kr67*[CaM-Ca], kf67=1.1 µM^{-1}min^{-1}, kr67=12 min^{-1}	Estimated from (13)
V86	Activated calmodulin binds calcineurin	Kf68*[CaM-Ca]*[CaN] – kr68*[CaM-Ca-CaN], kf68=3.1 µM^{-1}min^{-1}, kr68=7.3 min^{-1}	Fitted
V87	Activated calcineurin dephosphorylates NFAT	Vm29*[pNFAT]*([CaM-Ca-CaN]^n29/([CaM-Ca-CaN]^n29+kp29)), vm29=0.01044 min^{-1}, n29=7, kp29=3e-9 µM^7	Fitted
V88	Phosphorylation of NFAT in cytoplasm	Kf69*[NFAT], kf69=0.06 min^{-1}	Estimated from (14)
V89	NFAT moves from nucleus to cytoplasm	Kf70*[pNFAT$_N$], kf70=0.2 min^{-1}	Estimated from (14)
V90	Phosphorylation of NFAT in nucleus	Kf69*[NFAT$_N$]	Estimated from (14)
V91	NFAT moves in to nucleus	Kf71*[NFAT], kf71=3 min^{-1}	Fitted
V92	Synthesis of TGFβRII	Vm30, vm30=1.33e-5 µM/min	(12)
V93	Degradation of TGFβRII	Kf72*[TGFβR2], kf72=0.0278 min^{-1}	(12)

V94	TGFβ binds TGFβRII	Kf73*[TGFβ]*[TGFβR2] –kr73*[TLR], kf73=397 $\mu M^{-1} min^{-1}$, kr73=0.298 min^{-1}	(12)
V95	LR complex dimerize with TGFβRI receptor that activates SMAD1	Kf73*[TLR]*[TGFβR1$_{SMAD1}$] – kr73*[Dimer$_{SMAD1}$]	
V96	LR complex dimerize with TGFβRI receptor that activates SMAD2	Kf73*[TLR]*[TGFβR1$_{SMAD2}$] – kr73*[Dimer$_{SMAD2}$]	
V97	Internalization of dimer activating SMAD1	Kf66*[Dimer$_{SMAD1}$]	
V98	Internalization of dimer activating SMAD2	Kf66*[Dimer$_{SMAD2}$]	
V99	Dimer activating SMAD1 binds SMAD1	Kf74* [Dimer$_{SMAD1-INT}$]*[SMAD1] – kr74*[SMAD1-Dimer$_{SMAD1-INT}$], kf74=19000 $\mu M^{-1} min^{-1}$, kr74=0.971 min^{-1}	Fitted; (12)
V100	Dimer activating SMAD2 binds SMAD2	Kf74* [Dimer$_{SMAD2-INT}$]*[SMAD2] – kr74*[SMAD2-Dimer$_{SMAD2-INT}$]	
V101	Phosphorylation of SMAD1	Kf75*[SMAD1-Dimer$_{SMAD1-INT}$], kf75=44800 min^{-1}	(12)
V102	Phosphorylation of SMAD2	Kf75*[SMAD2-Dimer$_{SMAD2-INT}$]	
V103	Shuttling of phosphorylated SMAD1 into nucleus	Kf76*[pSMAD1], kf76=0.503 min^{-1}	(12)
V104	Shuttling of phosphorylated SMAD2 into nucleus	Kf76*[pSMAD2]	
V105	Phosphorylated SMAD1 binds SMAD4	Kf77*[pSMAD1]*[SMAD4] – kr77*[pSMAD1-SMAD4], kf77=3600 $\mu M^{-1} min^{-1}$, kr77=1460 min^{-1}	Fitted; (12)
V106	Phosphorylated SMAD2 binds SMAD4	Kf77*[pSMAD2]*[SMAD4] – kr77*[pSMAD2-SMAD4]	
V107	pSMAD1-SMAD4	Kf78*[pSMAD1-SMAD4], kf78=0.8 min^{-1}	(12)

	shuttling into nucleus		
V108	pSMAD2-SMAD4 shuttling into nucleus	Kf78*[pSMAD2-SMAD4]	
V109	SMAD4 shuttling into nucleus	Kf79*[SMAD4] – kr79*[$SMAD4_N$], kf79=0.0201 min^{-1}, kr79=0.174 min^{-1}	(12)
V110	pSMAD1 binds SMAD4 in nucleus	Kf80*[$SMAD4_N$]*[$pSMAD1_N$]-kr80*[pSMAD1-$SMAD4_N$], kf80=100 $\mu M^{-1} min^{-1}$, kr80=0.909 min^{-1}	(12)
V111	pSMAD2 binds SMAD4 in nucleus	Kf80*[$SMAD4_N$]*[$pSMAD2_N$]-kr80*[pSMAD2-$SMAD4_N$]	
V112	Dephosphorylation of pSMAD1-SMAD4 in nucleus	Kf81*[pSMAD1-$SMAD4_N$],kf81=0.05802 min^{-1}	Fitted
V113	Dephosphorylation of pSMAD2-SMAD4 in nucleus	Kf82*[pSMAD2-$SMAD4_N$], kf82=0.01104 min^{-1}	Fitted
V114	Dephosphorylation of pSMAD1 in nucleus	Kf81*[$pSMAD1_N$]	
V115	Dephosphorylation of pSMAD2 in nucleus	Kf82*[$pSMAD2_N$]	
V116	Dissociation of SMAD1-SMAD4 in nucleus	Kf83*[SMAD1-$SMAD4_N$], kf83=0.101 min^{-1}	(12)
V117	Dissociation of SMAD2-SMAD4 in nucleus	Kf83*[SMAD2-$SMAD4_N$]	
V118	Shuttling of SMAD1 into nucleus	Kf84*[SMAD1]-kr84*[$SMAD1_N$], kf84=0.162 min^{-1}, kr84=0.348 min^{-1}	(12)
V119	Shuttling of SMAD2 into nucleus	Kf84*[SMAD2]-kr84*[$SMAD2_N$]	
V120	Synthesis of SMAD1	Vm31, vm31=4.55e-5 µM/min	(12)
V121	Synthesis of SMAD2	Vm31	
V122	Degradation of SMAD1	Kf85*[SMAD1], kf85=8.46e-4 min^{-1}	Estimated from (15)

V123	Degradation of SMAD2	Kf85*[SMAD2]	
V124	Dissociation of receptor dimer that activates SMAD1	Kr66*[$Dimer_{SMAD1-INT}$]	
V125	Dissociation of receptor dimer that activates SMAD2	Kr66*[$Dimer_{SMAD2-INT}$]	
V126	Synthesis of receptor that activates SMAD1	Vm30	
V127	Synthesis of receptor that activates SMAD2	Vm30	
V128	Degradation of receptor that activates SMAD1	Kf72*[$TGF\beta R_{SMAD1}$]	
V129	Degradation of receptor that activates SMAD2	Kf72*[$TGF\beta R_{SMAD2}$]	
V130	R-SMADs activates SMAD7	Vm32*(vm33+[pSMAD1-$SMAD4_N$]^n32/(kp32+[pSMAD1-$SMAD4_N$]^n32)+[pSMAD2-$SMAD4_N$]^n33/(kp33+[pSMAD2-$SMAD4_N$]^n33)), vm32=0.005 µM/min, vm33=1e-4, n32=2, kp32=0.1 μM^2, n33=2, kp33=0.02 μM^2	Estimated from (6)
V131	Degradation of SMAD7 mRNA	Kf86*[mSMAD7], kf86=0.001 min^{-1}	Estimated from (3, 5)
V132	SMAD7 protein translation	Kf87*[mSMAD7], kf87=0.5 min^{-1}	Estimated from (3)
V133	Degradation of SMAD7 protein	Kf88*[SMAD7], kf88=0.00588 min^{-1}	Estimated from (3)
V134	SMAD7 promotes SMAD4 degradation	[SMAD4]*(vm34+vm35*[SMAD7]/([SMAD7]+kp35)), vm34=0.0012 min^{-1}, vm35=0.0035 min^{-1}, kp35=1 µM	Vm34 estimated from (3); fitted
V135	SMAD4 synthesis	Vm36, vm36=8.3056e-5 µM/min	(12)

V136	SMAD7 sequesters SMAD1-receptor complex	Kf89*[SMAD7]*[SMAD1-Dimer$_{SMAD1-INT}$]-kr89*[SMAD7-SMAD1-Dimer$_{SMAD1INT}$], kf89=300 $\mu M^{-1} min^{-1}$, kr89=0.024 min^{-1}	Estimated from (4)
V137	SMAD7 sequesters SMAD2-receptor complex	Kf89*[SMAD7]*[SMAD2-Dimer$_{SMAD2-INT}$]-kr89*[SMAD7-SMAD2-Dimer$_{SMAD2-INT}$]	
V138	Calcium outflux	[Ca]*kf90*[out-switch], kf90=0.03 min^{-1}, see calcium regulation below for details of [out-switch]	Fitted

Calcium regulation (rule-based):

[out-switch]$_0$=0 (unitless), [in-switch]$_0$=1 (unitless), [track]$_0$=0 (unitless);

[out-switch]=0, [in-switch]=1, [track]=0 if [Ca]<=0.025 μM

[out-switch]=([Ca]-0.025)*54, [in-switch]=2e-5, [track]=1 if ([Ca]>0.12 and [track]<1)

[out-switch]=([Ca]-0.025)*54, [in-switch]=2e-5, [track]=1 if ([Ca]>0.025 and [track]>0)

[out-switch]=([Ca]-0.025)*2000, [track]=0 if ([Ca]>0.025 and [track]<1)

Results of sample calcium kinetics are shown in S1_Fig.

S1_Table. Reaction descriptions, rules, reaction rates and kinetic parameters of TSP-1 model. Reactions are formulated based on experimental evidence in the literature. Reaction rates v# here match with the numbers in the model diagram provided in the article. Species$_N$ represents that the species is in the nucleus, other species, assumed in protein or miR form, are in the cytoplasm; mSpecies represents the mRNA of the species. The values of some rate constants are taken or estimated (and then optimized) from previous studies and experimental measurements as denoted by the Ref#. The values of all the other rate constants shown in this table are obtained after optimization against published EC data.

References

1. Li GW, Burkhardt D, Gross C, Weissman JS. Quantifying absolute protein synthesis rates reveals principles underlying allocation of cellular resources. Cell. 2014;157(3):624-35.
2. Qutub AA, Popel AS. A computational model of intracellular oxygen sensing by hypoxia-inducible factor HIF1 alpha. Journal of cell science. 2006;119(Pt 16):3467-80.
3. Schwanhausser B, Busse D, Li N, Dittmar G, Schuchhardt J, Wolf J, et al. Corrigendum: Global quantification of mammalian gene expression control. Nature. 2013;495(7439):126-7.
4. Yugandhar K, Gromiha MM. Protein-protein binding affinity prediction from amino acid sequence. Bioinformatics. 2014;30(24):3583-9.
5. Yang E, van Nimwegen E, Zavolan M, Rajewsky N, Schroeder M, Magnasco M, et al. Decay rates of human mRNAs: correlation with functional characteristics and sequence attributes. Genome research. 2003;13(8):1863-72.
6. Miller C, Schwalb B, Maier K, Schulz D, Dumcke S, Zacher B, et al. Dynamic transcriptome analysis measures rates of mRNA synthesis and decay in yeast. Molecular systems biology. 2011;7:458.
7. Yang X, Li H, Huang Y, Liu S. The dataset for protein-RNA binding affinity. Protein science : a publication of the Protein Society. 2013;22(12):1808-11.
8. Gantier MP, McCoy CE, Rusinova I, Saulep D, Wang D, Xu D, et al. Analysis of microRNA turnover in mammalian cells following Dicer1 ablation. Nucleic acids research. 2011;39(13):5692-703.
9. Klironomos FD, Berg J. Quantitative analysis of competition in posttranscriptional regulation reveals a novel signature in target expression variation. Biophysical journal. 2013;104(4):951-8.
10. Gokhale SA, Gadgil CJ. Analysis of miRNA regulation suggests an explanation for 'unexpected' increase in target protein levels. Molecular bioSystems. 2012;8(3):760-5.
11. Riley KJ, Yario TA, Steitz JA. Association of Argonaute proteins and microRNAs can occur after cell lysis. Rna. 2012;18(9):1581-5.
12. Nicklas D, Saiz L. Computational modelling of Smad-mediated negative feedback and crosstalk in the TGF-beta superfamily network. Journal of the Royal Society, Interface / the Royal Society. 2013;10(86):20130363.
13. Crouch TH, Klee CB. Positive cooperative binding of calcium to bovine brain calmodulin. Biochemistry. 1980;19(16):3692-8.
14. Shen T, Cseresnyes Z, Liu Y, Randall WR, Schneider MF. Regulation of the nuclear export of the transcription factor NFATc1 by protein kinases after slow fibre type electrical stimulation of adult mouse skeletal muscle fibres. The Journal of physiology. 2007;579(Pt 2):535-51.
15. Lo RS, Massague J. Ubiquitin-dependent degradation of TGF-beta-activated smad2. Nature cell biology. 1999;1(8):472-8.
