# Supplementary material for: Transcriptional and Post-Transcriptional Regulation of Thrombospondin-1 Expression: A Computational Model
Source: PLoS Comput Biol. 2017 Jan 3;13(1):e1005272. doi: 10.1371/journal.pcbi.1005272 (PMC5207393; doi:10.1371/journal.pcbi.1005272)
Supplement: S2 Table — (PDF) [file pcbi.1005272.s003.pdf]

S2_Table: Differential equations and species initial conditions

Species	Differential Equations ($\frac{d[\text{Species}]}{dt}$)	Species Initial Condition (μM)
	**Intracellular TSP-1 regulation (Subpart A)**	
HIF1,2α	v1-v2-v3-v7	0.2077, 0.2958
HIF1,2α-FIH complex	v3-v11	6.808e-4, 0.2532
HIF1,2α-PHD complex	v7-v12	0.0158, 0.0225
FIH-O_2-Fe-DG	v4+v11-v3	0.8824
O_2	-v4-v8	Nx(21% O_2):209, Hx (2% O_2):19.9
FIH-DG-Fe	v5-v4	0.2712
DG	-v5-v9	999
FIH-Fe	v6-v5	8.73e-3
Fe	-v6-v10	49.6
FIH	-v6	4.396e-4
HIF1,2α/OH	v11+v12-v13	0.00148, 0.0047
VHL	v14-v13	1.116
HIF1,2α/OH-VHL	v13-v14	0.03011, 0.00471
PHD2-O_2-Fe-DG	v12+v8-v7	0.789
PHD2-Fe-DG	v9-v8	0.949
PHD2-Fe	v10-v9	0.0569
PHD2	-v10	2.29e-3
HIF1α_N	v2-v18	0.0577
HIF2α_N	v2-v19	0.0821
HIF1β_N	-v18-v19	0.8756
TTP	v17-v16	0.0700
mTTP	V22-v21	0.00127
HIF1-dimer$_N$	v18	0.0101
HIF2-dimer$_N$	v19	0.0144
MYC	v20-v30-v15	0.0161
MYC$_N$	v30	0.0322
mMXI1	v24-v25	9.36e-5
MXI1	v26-v27-v28	0.0234
MXI1$_N$	v28	0.0585
mLin28B	V31-v79	1.88e-5
Lin28B	V33-v80-v34	0.00113
Lin28B$_N$	V34-v35	0.0113
Lin28B$_N$-pri-let7$_N$	V35	0.0591
mPSAP	V32-v36	3.36e-5
PSAP	V37-v38-v39	0.03357
PSAP$_N$	V39	0.0839
mP53	V40-v41	6.58e-5
P53	V42-v43-v44	0.1468
P53$_N$	V44	0.0587
Pri-miR-18a	V29-v45	9.54e-4
Pre-miR-18a	V45-v46-v47	7.91e-6

miR-18a	V47-v48-v49	0.00221
miR-18a RISC	V49-v50+v51	0.00288
miR-18a RISC-mTSP1	V50-v51	0
mTSP1/p-body	V51-v52-v53	3.92e-5
Pri-let-7_{N}	v23-v54	6.55e-5
Pre-let-7	V54-v55-v56	5.76e-6
Let-7	V56-v61-v62	2.506e-4
AGO1	V60-v57-v62-v49	0.7767
Let-7 RISC	V62-v63-v69+v64+v70	0.00278
mAGO1/p-body	V64-v65-v66	0.01795
mAGO1	V59-v58-v63+v66	0.00127
Let-7 RISC-mAGO1	V63-v64	6.29e-5
mDicer	V67-v68-v69+v72	2.93e-3
Let-7 RISC-mDicer	V69-v70	2.98e-5
mDicer/p-body	V70-v71-v72	0.0265
Dicer	V73-v74	1.0476
mTSP1	V75-v77-v50+v53	2.36e-5
TSP1	V76-v78	0.00867
	**TGFβ activation of TSP-1 (Subpart B)**	
TGFβ$R1_{SMAD1}$	V124+v126-v81-v95-v128	4.78e-4
TGFβ$R1_{SMAD1-INT}$	V81	0.0048
TGFβ$R1_{SMAD2}$	V127+v125-v96-v129-v82	4.78e-4
TGFβ$R1_{SMAD2-INT}$	V82	0.0048
TGFβR2	V92+v124+v125-v93-v94-v83	4.78e-4
TGFβ$R2_{INT}$	V83	0.0048
TGFβ	-v94	0
TLR	V94-v95-v96	0
$Dimer_{SMAD1}$	V95-v97	0
$Dimer_{SMAD2}$	V96-v98	0
$Dimer_{SMAD1-INT}$	V97-v99+v101	0
$Dimer_{SMAD2-INT}$	V98-v100+v102	0
SMAD1- $Dimer_{SMAD1-INT}$	V99-v101	0
SMAD2- $Dimer_{SMAD2-INT}$	V100-v102	0
SMAD1	V120-v122-v99-v128	0.0538
SMAD2	V121-v123-v100-v129	0.0538
pSMAD1	V101-v103-v105	0
pSMAD2	V102-v104-v106	0
SMAD4	V135-v134-v105-v106-v109	0.0619
pSMAD1-SMAD4	V105-v107	0
pSMAD2-SMAD4	V106-v108	0
$pSMAD1_{N}$	V103-v114-v110	0
$pSMAD2_{N}$	V104-v115-v111	0
$SMAD4_{N}$	V109-v110-v111+v116+v117	0.0072
pSMAD1-$SMAD4_{N}$	V110-v112	0
pSMAD2-$SMAD4_{N}$	V111-v113	0
SMAD1-$SMAD4_{N}$	V112-v116	0

SMAD2-SMAD4$_N$	V113-v117	0
SMAD1$_N$	V114+v116+v118	0.025
SMAD2$_N$	V115+v117+v119	0.025
mSMAD7	V130-v131	5e-4
SMAD7	V132-v133-v136-v137	0.0425
SMAD7-SMAD1-Dimer$_{SMAD1-INT}$	V136	0
SMAD7-SMAD2-Dimer$_{SMAD2-INT}$	V137	0
Ca	V84-v85-v138, see calcium regulation in S1_Table	0.0254
CaM	-V85	5.9233
CaM-Ca	V85-v86	0.0138
CaN	-v86	4.9388
CaM-Ca-CaN	V86	0.0289
pNFAT	V89+v88-v87	1.5022
NFAT	V87-v88-v91	2.87e-5
NFAT$_N$	V91-v90	0.001435
pNFAT$_N$	V90-v89	4.31e-4

S2_Table. Model differential equations and species initial conditions of TSP-1 model. Initial conditions here refer to the steady-state (normoxia) concentration of each species in ECs without TGFβ treatment. To maintain a moderate complexity, the model assumes that transcription factors or enzymes in Hill-type (Michaelis-Menten) reactions are unconsumed, and mRNAs are unconsumed in translation. Values of initial conditions used in the model (e.g. oxygen sensing, TGFβ signaling) are either estimated (following by optimization against published EC data) or taken from literature (1, 2).

References

1. Nicklas D, Saiz L. Computational modelling of Smad-mediated negative feedback and crosstalk in the TGF-beta superfamily network. Journal of the Royal Society, Interface / the Royal Society. 2013;10(86):20130363.
2. Qutub AA, Popel AS. A computational model of intracellular oxygen sensing by hypoxia-inducible factor HIF1 alpha. Journal of cell science. 2006;119(Pt 16):3467-80.
