# Supplementary material for: Transcriptional and Post-Transcriptional Regulation of Thrombospondin-1 Expression: A Computational Model
Source: PLoS Comput Biol. 2017 Jan 3;13(1):e1005272. doi: 10.1371/journal.pcbi.1005272 (PMC5207393; doi:10.1371/journal.pcbi.1005272)
Supplement: S1 Fig — (PDF) [file pcbi.1005272.s004.pdf]

S1_Fig

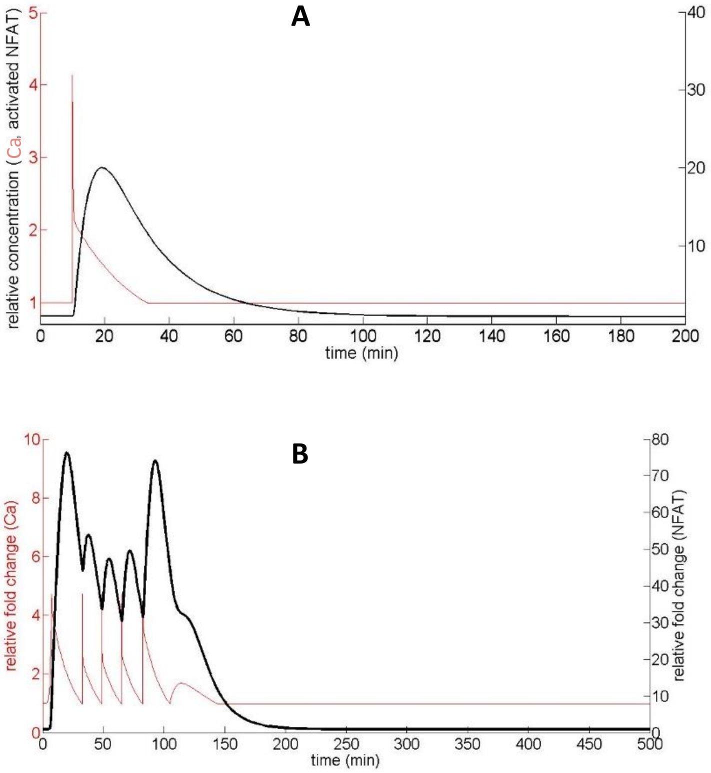

S1_Fig. Model of TGFβ-induced calcium regulation and downstream activation of NFAT in ECs. Multiple rule-based reactions are responsible for the TGFβ-induced calciumn regulation in the model. An assumption is that rate of calcium influx is dependent on the concentration of internalized TGFβ receptors that have bound ligands. The rules define that when the intracellular free calcium concentration reaches a threshold (~120 nM), calcium influx is significantly reduced and outflux rate is increased. The outflux rate is proportional to the difference between current intracelluar free calcium concentration and the baseline concentartion. When the intracelluar free calcium concentration is below the baseline level, rate of calcium influx and outflux is restored. The mathematical equations defining the rules are shown in S1_Table. (A-B) The resulting calcium profiles (red curve) in response to TGFβ treatment display a periodic behavior. Simulated NFAT (activated) dynamics (black curves, normalized in terms of relative fold changes) exhibit a stair-case like behavior which resembles the experimental data measured in (1).

References

1. Noren DP, Chou WH, Lee SH, Qutub AA, Warmflash A, Wagner DS, et al. Endothelial cells decode VEGF-mediated Ca2+ signaling patterns to produce distinct functional responses. Science signaling. 2016;9(416):ra20.
