# Supplementary material for: Transcriptional and Post-Transcriptional Regulation of Thrombospondin-1 Expression: A Computational Model
Source: PLoS Comput Biol. 2017 Jan 3;13(1):e1005272. doi: 10.1371/journal.pcbi.1005272 (PMC5207393; doi:10.1371/journal.pcbi.1005272)
Supplement: S2 Fig — (PDF) [file pcbi.1005272.s005.pdf]

S2_Fig

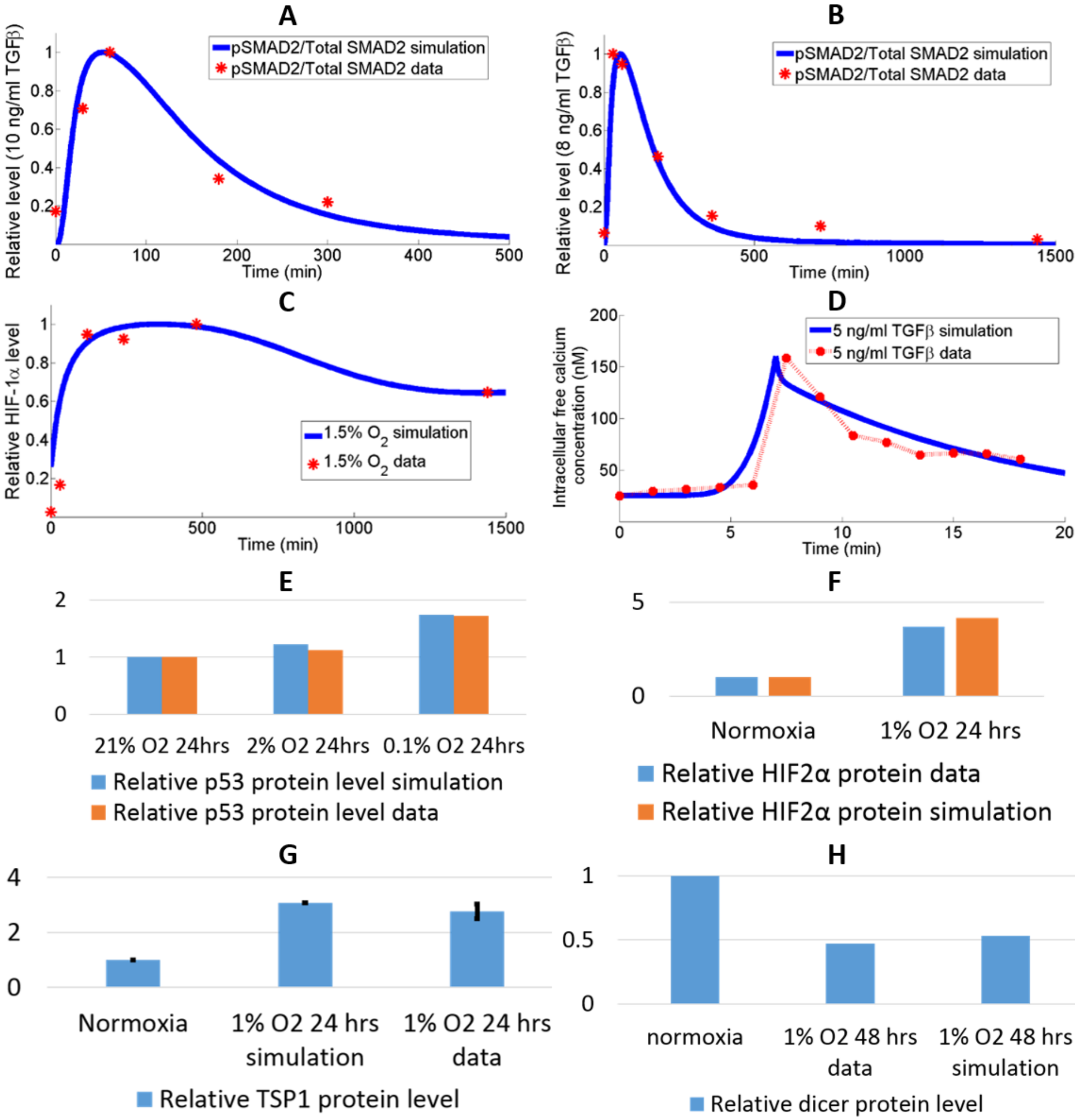

A
pSMAD2/Total SMAD2 simulation
pSMAD2/Total SMAD2 data
Relative level (10 ng/ml TGFβ)
Time (min)
B
pSMAD2/Total SMAD2 simulation
pSMAD2/Total SMAD2 data
Relative level (8 ng/ml TGFβ)
Time (min)
C
1.5% O2 simulation
1.5% O2 data
Relative HIF-1α level
Time (min)
D
5 ng/ml TGFβ simulation
5 ng/ml TGFβ data
Intracellular free calcium concentration (nM)
Time (min)
E
21% O2 24hrs
2% O2 24hrs
0.1% O2 24hrs
Relative p53 protein level simulation
Relative p53 protein level data
F
Normoxia
1% O2 24 hrs
Relative HIF2α protein data
Relative HIF2α protein simulation
G
Normoxia
1% O2 24 hrs simulation
1% O2 24 hrs data
Relative TSP1 protein level
H
normoxia
1% O2 48 hrs data
1% O2 48 hrs simulation
Relative dicer protein level

S2_Fig. Additional model calibration against published fibroblast data using different paramter values. Simulation and data of (A) relative ratio of phosphorylated SMAD2 protein in response to 10 ng/ml TGFβ1 in human lung myofibroblasts (1), (B) relative ratio of phosphorylated SMAD2 protein in response to 8 ng/ml TGFβ1 in human normal primary fibroblasts (2), (C) relative level of HIF1α protein in 1.5% O_2 in human embryonic fibroblasts (3), (D) concentrations of intracelluar free calcium in response to 5 ng/ml TGFβ1 treatment over twenty minutes (4), (E) relative p53 protein levels in different oxygen tensions in normal human lung fibroblasts (5), (F-G) relative HIF2α and TSP-1 protein levels in hypoxia (1% O_2) in pulmonary-derived murine fibroblasts (6), (H) relative Dicer protein level in response to hypoxia (1% O_2) in mouse embryonic fibroblasts (7). (A-H) The primary goal of these additional validations is to show that our model, which is originally based in ECs, has the potential to be applied to simulate TSP-1 pathway signals in fibroblasts. Aiming for the minimum amount of changes in the EC paramter set, values of three parameters are altered here in order to fit the data in fibroblasts: kf74 is set to 1200 $\mu M^{-1} min^{-1}$, maximum intracelluar calcium allowed is set to 0.16 μM, and kp1 is set to 0.023 μM^4.

References

1. Roach KM, Feghali-Bostwick C, Wulff H, Amrani Y, Bradding P. Human lung myofibroblast TGFbeta1-dependent Smad2/3 signalling is Ca(2+)-dependent and regulated by KCa3.1 K(+) channels. Fibrogenesis & tissue repair. 2015;8:5.
2. Li Q, Zhang D, Wang Y, Sun P, Hou X, Larner J, et al. MiR-21/Smad 7 signaling determines TGF-beta1-induced CAF formation. Scientific reports. 2013;3:2038.
3. Poulios E, Trougakos IP, Gonos ES. Comparative effects of hypoxia on normal and immortalized human diploid fibroblasts. Anticancer research. 2006;26(3A):2165-8.
4. Alevizopoulos A, Dusserre Y, Ruegg U, Mermod N. Regulation of the transforming growth factor beta-responsive transcription factor CTF-1 by calcineurin and calcium/calmodulin-dependent protein kinase IV. The Journal of biological chemistry. 1997;272(38):23597-605.
5. Mizuno S, Bogaard HJ, Voelkel NF, Umeda Y, Kadowaki M, Ameshima S, et al. Hypoxia regulates human lung fibroblast proliferation via p53-dependent and -independent pathways. Respiratory research. 2009;10:17.
6. Labrousse-Arias D, Castillo-Gonzalez R, Rogers NM, Torres-Capelli M, Barreira B, Aragones J, et al. HIF-2alpha-mediated induction of pulmonary thrombospondin-1 contributes to hypoxia-driven vascular remodelling and vasoconstriction. Cardiovascular research. 2016;109(1):115-30.
7. Rupaimoole R, Wu SY, Pradeep S, Ivan C, Pecot CV, Gharpure KM, et al. Hypoxia-mediated downregulation of miRNA biogenesis promotes tumour progression. Nature communications. 2014;5:5202.
