# Supplementary material for: Transcriptional and Post-Transcriptional Regulation of Thrombospondin-1 Expression: A Computational Model
Source: PLoS Comput Biol. 2017 Jan 3;13(1):e1005272. doi: 10.1371/journal.pcbi.1005272 (PMC5207393; doi:10.1371/journal.pcbi.1005272)
Supplement: S3 Fig — (PDF) [file pcbi.1005272.s006.pdf]

S3_Fig

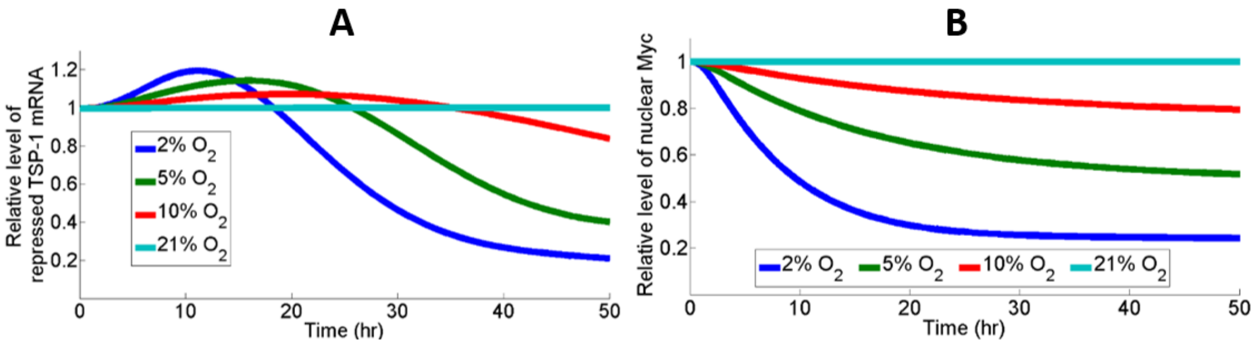

S3_Fig. De-suppression of TSP-1 mRNA and downregulation of Myc in hypoxia. In response to hypoxic stresses, the model predicts (A) a significant reduction in the amount of TSP-1 mRNAs that are suppressed by miRs. The initial rises in the simulation curves are due the rapid increase in hypoxia-driven TSP-1 transcriptional activation and the delayed, gradual downregulation of miR-18a. (B) Hypoxia promotes Myc protein degradation and results in its downregulation. Decrease in the abundance of Myc leads to a drop in the expression of miR-18a because of reduced transcriptional activation.
