# Supplementary material for: Transcriptional and Post-Transcriptional Regulation of Thrombospondin-1 Expression: A Computational Model
Source: PLoS Comput Biol. 2017 Jan 3;13(1):e1005272. doi: 10.1371/journal.pcbi.1005272 (PMC5207393; doi:10.1371/journal.pcbi.1005272)
Supplement: S4 Fig — (PDF) [file pcbi.1005272.s007.pdf]

S4_Fig

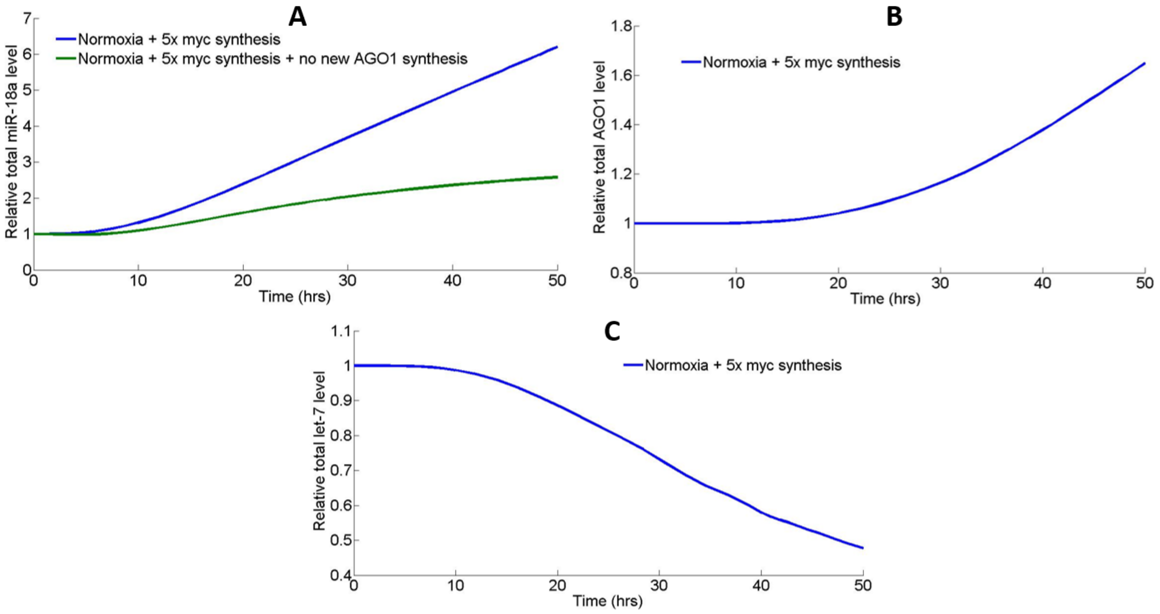

S4_Fig. AGO1 upregulation following myc overexpression. (A) Hyperactive Myc activity results in an increased level of miR-18a, which is partially contributed by (B) an increase in AGO1 level. (C) AGO1 upregulation is a result of let-7 downregulation by the Myc-Lin28B-let7 axis.
