# Supplementary material for: Transcriptional and Post-Transcriptional Regulation of Thrombospondin-1 Expression: A Computational Model
Source: PLoS Comput Biol. 2017 Jan 3;13(1):e1005272. doi: 10.1371/journal.pcbi.1005272 (PMC5207393; doi:10.1371/journal.pcbi.1005272)
Supplement: S5 Fig — (PDF) [file pcbi.1005272.s008.pdf]

**S5_Fig**

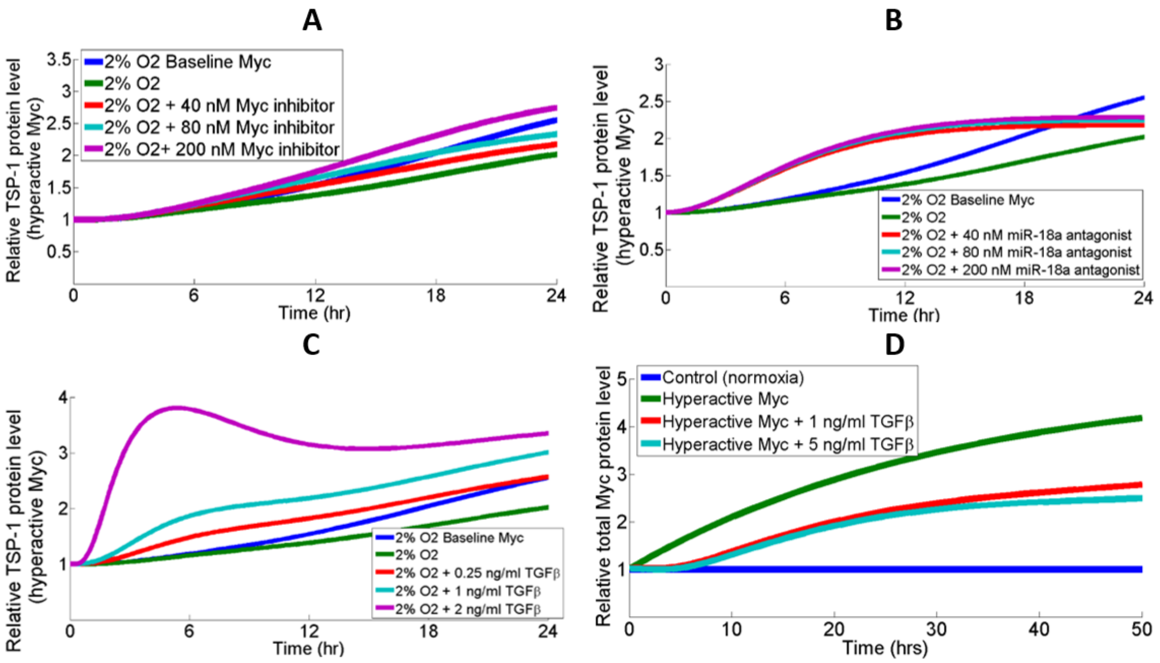


**S5_Fig. Testing different therapeutic strategies in hypoxia and hyperactive Myc conditions.** Simulations of TSP-1 protein expression in response to different doses of (A) Myc inhibitor, (B) miR-18a antagonist, and (C) TGFβ under the condition of Myc hyperactivity (Myc synthesis rate multiplied by 5) and hypoxia (2% Oxygen). Unlike the results presented in Fig 7, here TGFβ stimulation is predicted to be more effective compared to the other two strategies throughout a 24-hour simulation timespan, which agrees with the sensitivity analysis results shown in Fig 9. (D) TGFβ treatment not only induces TSP-1 transcription directly but also downregulates Myc expression in scenarios of hyperactive Myc (control is baseline Myc, and simulations are in normoxia). (A-D) Results are normalized with respect to the normoxic steady state value computed with baseline Myc activity.
