# Supplementary material for: Transcriptional and Post-Transcriptional Regulation of Thrombospondin-1 Expression: A Computational Model
Source: PLoS Comput Biol. 2017 Jan 3;13(1):e1005272. doi: 10.1371/journal.pcbi.1005272 (PMC5207393; doi:10.1371/journal.pcbi.1005272)
Supplement: S6 Fig — (PDF) [file pcbi.1005272.s009.pdf]

**S6_Fig**

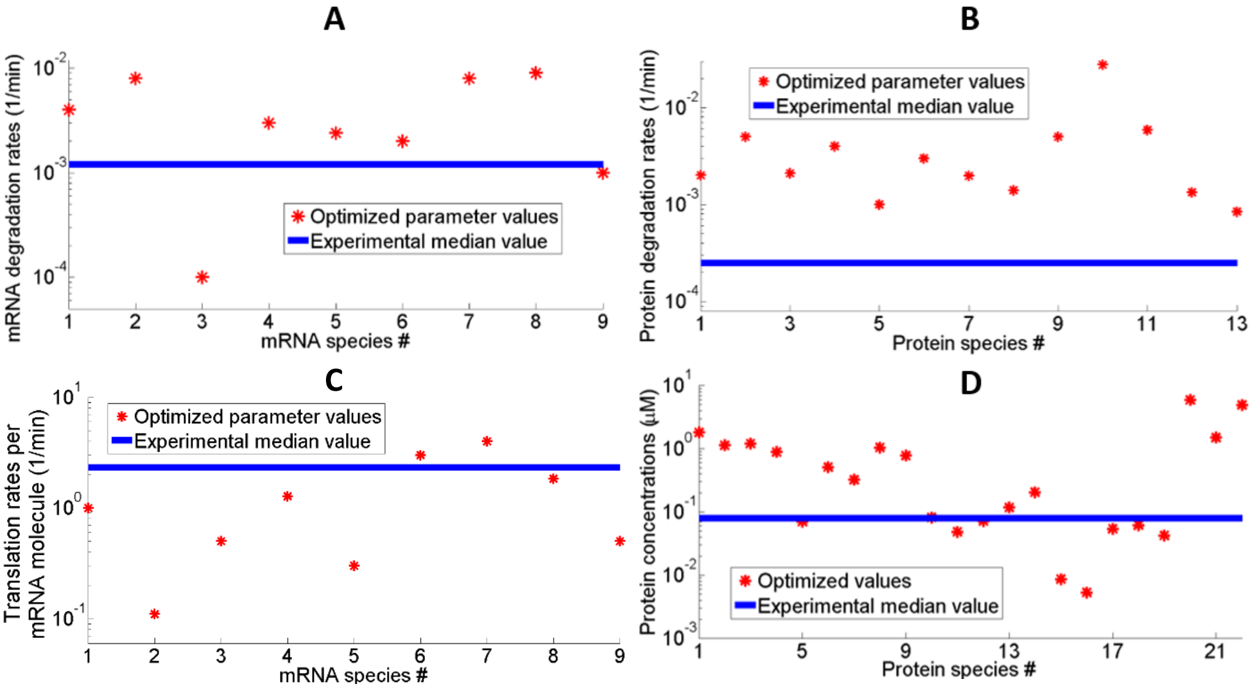


**S6_Fig. Model parameter distributions.** Distribution of protein/mRNA turnover parameters and initial conditions used in the model including (A) mRNA degradation rates of different species (median 1.2e-3 $min^{-1}$), (B) protein degradation rates of different species (median 2.5e-4 $min^{-1}$), (C) rate of translation of different mRNA species (median 2.33 proteins per mRNA per min), and (D) protein concentrations of different species (median 0.08 µM). (A-D) The red asterisks are optimized model parameter values and the blue lines are the experimental median values obtained from literature (1, 2).

## References


1. Yang E, van Nimwegen E, Zavolan M, Rajewsky N, Schroeder M, Magnasco M, et al. Decay rates of human mRNAs: correlation with functional characteristics and sequence attributes. Genome research. 2003;13(8):1863-72.
2. Schwanhausser B, Busse D, Li N, Dittmar G, Schuchhardt J, Wolf J, et al. Corrigendum: Global quantification of mammalian gene expression control. Nature. 2013;495(7439):126-7.
