# Supplementary material for: Transcriptional and Post-Transcriptional Regulation of Thrombospondin-1 Expression: A Computational Model
Source: PLoS Comput Biol. 2017 Jan 3;13(1):e1005272. doi: 10.1371/journal.pcbi.1005272 (PMC5207393; doi:10.1371/journal.pcbi.1005272)
Supplement: S7 Fig — (PDF) [file pcbi.1005272.s010.pdf]

S7_Fig

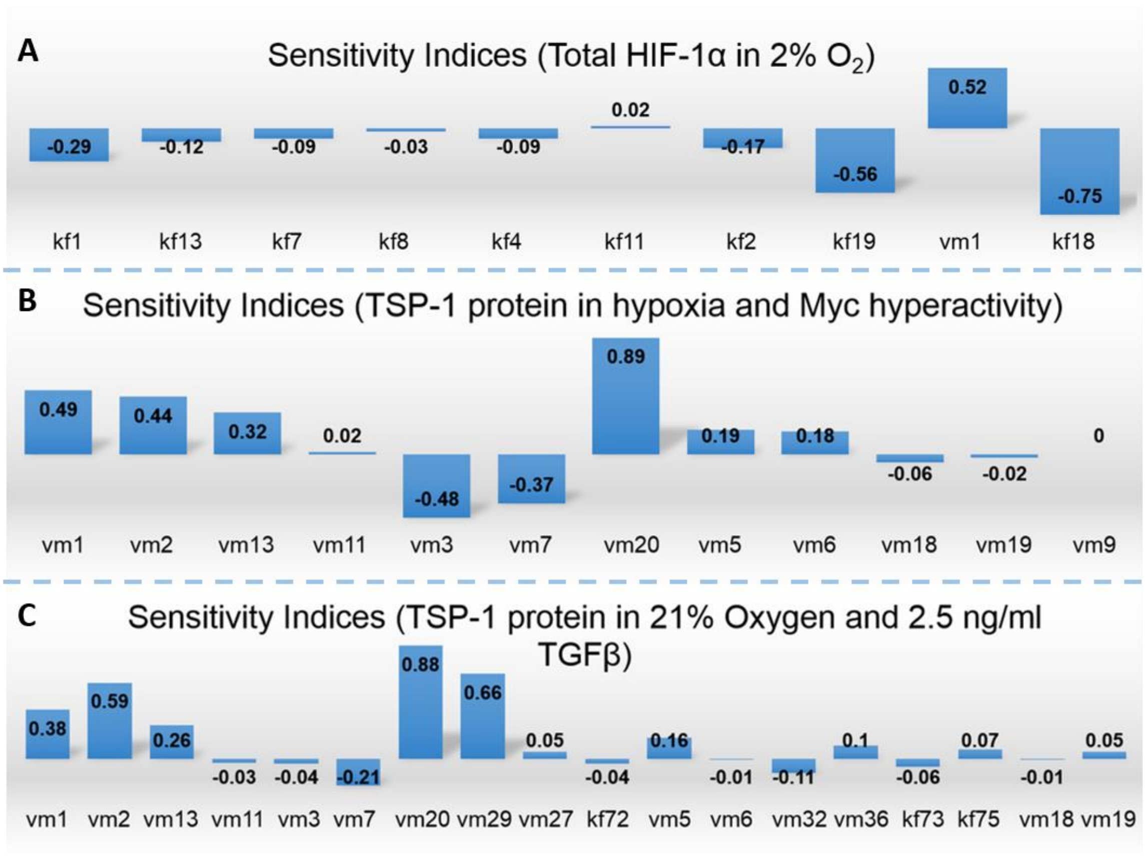

S7_Fig. Additional sensitivity analysis. AUCs of (A) total HIF-1α in 48 hours and (B-C) TSP-1 protein in 24 hours under different simulated conditions. (A-C) Rate descriptions—kf1: HIF-1α translocation into nucleus; kf2: HIF-1α binds FIH complex; kf4: oxygen binds FIH complex; kf7: HIF-1α binds PHD complex; kf8: oxygen binds PHD complex; kf11: HIF1α-OH-FIH dissociation; kf13: HIF1α-OH-PHD dissociation; kf18: TTP synthesis; kf19: HIF-1α binds HIF-1β; vm1: HIF-1α synthesis; vm2: HIF-2α synthesis; vm3: Myc synthesis; vm5: let-7 synthesis; vm6: MXI-1 synthesis; vm7: miR-18a synthesis; vm9: LIN28B synthesis; vm11: PSAP synthesis; vm13: p53 synthesis; vm18: Ago1 synthesis; vm19: Dicer synthesis; vm20: TSP-1 synthesis; kf72: TGFβR degradation; kf73: TGFβ binds its receptor; kf75: R-SMAD phosphorylation; vm27: TGFβ-mediated calcium influx; vm29: NFAT dephosphorylation; vm32: SMAD7 synthesis; vm36: SMAD4 synthesis.
